# Antibody discovery identifies regulatory mechanisms of protein arginine deiminase 4

**DOI:** 10.1038/s41589-023-01535-8

**Published:** 2024-02-02

**Authors:** Xin Zhou, Sophie Kong, Allison Maker, Soumya G. Remesh, Kevin K. Leung, Kliment A. Verba, James A. Wells

**Affiliations:** 1 Department of Pharmaceutical Chemistry, University of California San Francisco, San Francisco, CA USA; 2 Department of Cellular and Molecular Pharmacology, University of California San Francisco, San Francisco, CA USA; 3 Present Address: Department of Cancer Biology, Dana-Farber Cancer Institute, Boston, MA USA; 4 Present Address: Department of Biological Chemistry and Molecular Pharmacology, Harvard Medical School, Boston, MA USA

**Keywords:** Immunology, Structural biology, Chemical tools, Enzymes

## Abstract

Unlocking the potential of protein arginine deiminase 4 (PAD4) as a drug target for rheumatoid arthritis requires a deeper understanding of its regulation. In this study, we use unbiased antibody selections to identify functional antibodies capable of either activating or inhibiting PAD4 activity. Through cryogenic-electron microscopy, we characterized the structures of these antibodies in complex with PAD4 and revealed insights into their mechanisms of action. Rather than steric occlusion of the substrate-binding catalytic pocket, the antibodies modulate PAD4 activity through interactions with allosteric binding sites adjacent to the catalytic pocket. These binding events lead to either alteration of the active site conformation or the enzyme oligomeric state, resulting in modulation of PAD4 activity. Our study uses antibody engineering to reveal new mechanisms for enzyme regulation and highlights the potential of using PAD4 agonist and antagonist antibodies for studying PAD4-dependency in disease models and future therapeutic development.

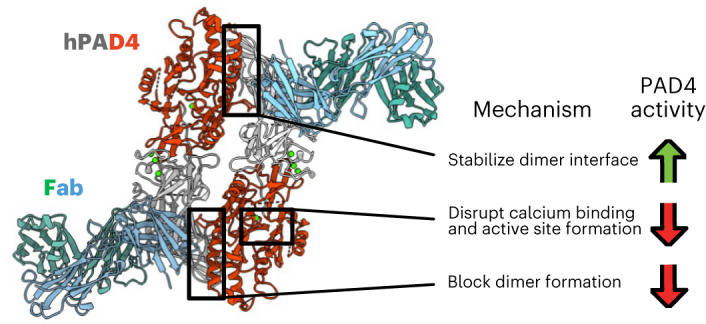

## Abstract (web summary)

Unbiased antibody discovery identified allosteric regulators of PAD4, revealing mechanisms to alter PAD4’s activity and providing tools to study rheumatoid arthritis.

## Main

Protein arginine deiminase 4 (PAD4) is a calcium-dependent enzyme that catalyzes hydrolysis of peptidyl arginine sidechains to citrulline in proteins^1–3^. In neutrophils, PAD4-catalyzed chromatin decondensation plays a crucial yet ambiguous role in inducing an inflammatory form of neutrophil cell death called NETosis. Multiple reports have shown a correlation between PAD4 expression and histone citrullination leading to NETosis, but the link between citrullination and NET formation is still largely complicated^4–7^. During this process, PAD4 and other intracellular contents are released to the extracellular space where PAD4 can create extracellular citrullinated neoepitopes. These neoepitopes are recognized by the immune system and further trigger inflammatory diseases such as rheumatoid arthritis (RA)^8,9^. Small-molecule PAD4 inhibitors were developed and found to be effective in alleviating RA phenotypes in mouse models, indicating the relevance of PAD4 in RA pathology^10,11^. While small-molecule PAD4 inhibitors show promise in preclinical studies of targeting RA, they often lack specificity and potency due to direct targeting of the enzyme’s active site that is highly conserved across PAD isoforms^12–14^.

Antibodies are powerful tools for capturing the dynamic nature of proteins by binding to both enzyme active sites and allosteric sites and interrogating different protein conformations^15^. For example, deliberately selecting for antibodies to natural on- or off-states in proteins can be achieved by first ‘trapping’ those conformations and then using differential in vitro antibody selection to isolate state-specific binders^16–18^. Instead of trapping the enzymatic state prior to selection, we hypothesized that using unbiased antibody selections on a native enzyme sampling various conformations in solution will allow us to identify new conformations that result in an active or inactive enzyme. PAD4 is ideally suited for such an approach to better understand the mechanisms and protein states associated with activation and inhibition of the enzyme. Moreover, antibodies are an ideal modality for targeting extracellular PAD4 since, unlike small molecules, they do not penetrate cells and would not interfere with normal homeostatic activities of intracellular PAD enzymes.

Structural studies show each PAD4 binds five Ca^2+^ ions in two different pockets to become fully activated, and calcium-bound PAD4 is 10,000 times as active as apo-PAD4 (refs. ^19,20^). PAD4’s calcium-dependent activation may be relevant when released into extracellular milieu where calcium concentration reaches millimolar (mM) levels compared to micromolar (µM). However, its extracellular regulation remains ambiguous given the high oxidizing environment outside cells^21^. Also contributing to PAD4 extracellular regulation is the presence of autoantibodies, found in 20–45% of RA patients, that bind and further activate PAD4. The presence of these autoantibodies is typically associated with a more severe and erosive disease phenotype^22–27^. The binding epitopes of several patient-derived anti-PAD4 autoantibodies have been characterized, but their mechanisms of action remain unclear^26,27^. The existence of endogenous anti-PAD4 antibodies lead us to believe that we can use in vitro methods to identify anti-PAD4 antibodies also capable of modulating extracellular PAD4 activity.

Here, we used an unbiased antibody selection strategy, coupled with functional screening and cryogenic-electron microscopy (cryo-EM) structural analysis, to identify new conformations and mechanisms for inhibiting or activating both murine and human PAD4 in the presence of high Ca^2+^. We discovered that PAD4 activity can be enhanced through antibody binding to an interface loop that promotes PAD4 dimerization while reducing disorder in the substrate-binding loop. We also discovered an inhibitory antibody that binds and re-structures a helix in the Ca^+2^ binding pocket that mediates a conformational change in the active site, preventing calcium ion and substrate binding. These engineered antibodies form a versatile toolkit for studying PAD4-dependence of disease states in both mouse models and patient samples. Through structural analysis of the antibody–PAD4 complexes, we shed light on previously unknown PAD4 regulatory mechanisms, providing new opportunities for pharmacological targeting of the enzyme.

## Results

### Identifying PAD4 antibodies by phage display

**Extended Data Fig. 1:**
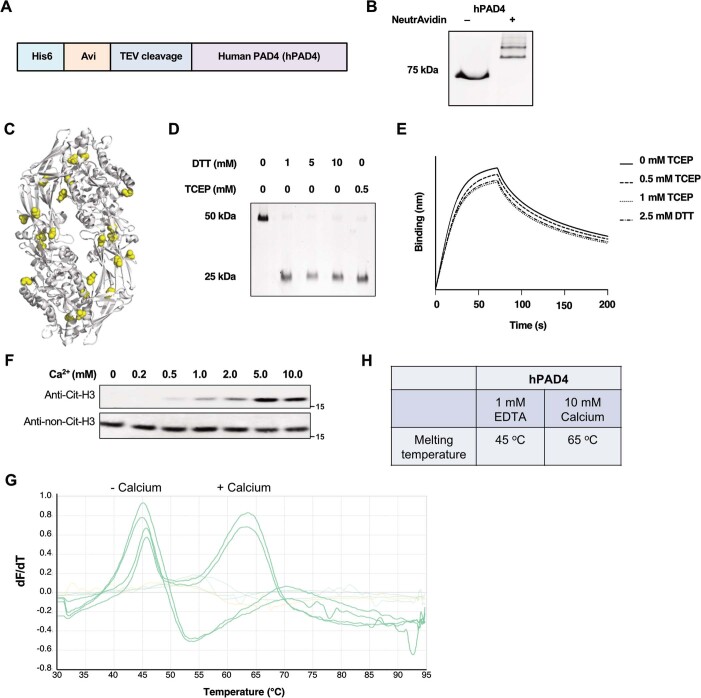
Expression and biophysical characterization of hPAD4. (**a**) Map of hPAD4 gene expressed in C43 or BL21 *E. coli*. N-terminal 6x His tag and Avi tag are separated by a protease cleavage site to hPAD4 to remove tags. (**b**) Gel shift assay of full length hPAD4 (left, 74 kDa) and biotinylated hPAD4 bound to NeutrAvidin (right, 134 kDa). (**c**) PAD4 enzyme contains 19 free cysteines (colored in yellow), requiring storage with reducing agent to prevent oxidation of cysteines. (**d**) SDS–PAGE showing reduction of the inter-heavy and light chain disulfide of a 4G10^4D5^ antibody upon addition of reducing agents. The 4G10^4D5^ antibody adopts the antibody scaffold of the Fab-phage library used for PAD4 antibody selection. 50 kDa band represents disulfide-linked Fab while 25 kDa bands represent separated heavy and light chains following TCEP reduction. (**e**) Biolayer Interferometry (BLI) showing binding of Fab 4G10^4D5^ to its cognate antibody is not influenced by various reducing agents. (**f**) Immunoblotting detection of PAD4-mediated Histone 3 citrullination (Cit-H3) showing PAD4 activity is calcium dependent. (**g**) Differential scanning fluorimetry (DSF) plot showing melting temperatures of hPAD4 in the presence and absence of Ca^2+^. (**h**) Table listing PAD4 melting temperatures measured by DSF. Source data

As a first step toward antibody generation, it was critical to produce highly purified native forms of PAD4 suitable for in vitro phage antibody selections. N-terminal biotinylated forms of human PAD4 (hPAD4) and mouse PAD4 (mPAD4) were constructed and expressed in BirA-expressing *Escherichia coli* for facile one-step purification and immobilization on streptavidin magnetic beads for phage selection (Extended Data Fig. 1a). Biotinylation was validated by a gel shift assay (Extended Data Fig. 1b). Because PAD4 has free cysteines on the surface, 0.5 mM of Tris(2-carboxyethyl)phosphine (TCEP) was supplemented to the protein purification and phage selection buffers to prevent protein aggregation and loss of function (Extended Data Fig. 1c). As antibodies are held together by a series of disulfide bonds, we confirmed that the Fab scaffold used in this phage library does not lose the antigen-binding capability even in the presence of the TCEP needed to stabilize PAD4 (Extended Data Fig. 1d,e).

**Fig. 1:**
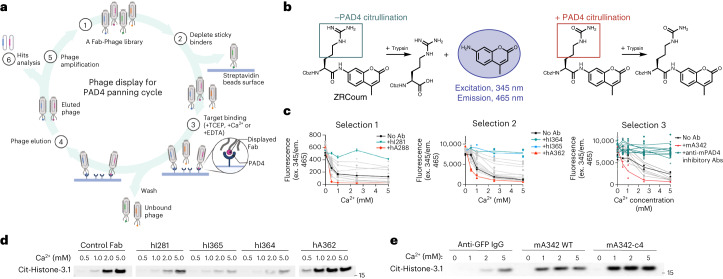
PAD4–antibody selection and hits characterization. **a**, PAD4 phage display schematic. The Fab-phage library (1) was depleted of nonspecific streptavidin binders (2); the remaining soluble Fab-phage were allowed to bind to PAD4 immobilized on streptavidin in the presence of 1 mM Ca^2+^ or 1 mM EDTA with TCEP and washed (3); the bound Fab-phage were eluted (4), amplified (5) and subjected to additional selection rounds before final characterization of individual hits (6). **b**, Schematic of fluorescent-substrate PAD4 activity assay (derived from Sabulski et al.). Higher fluorescent signal is indicative of lower PAD4 activity. **c**, Characterization of Fab binding effect on PAD4 activity from hPAD4 selection 1 and 2 and mPAD4 selection 4 (Extended Data Fig. 3a) via fluorescent-substrate activity assay. Highlighted clones hI281, hA288, hA362, hI364, hI365 and mA342 are described in the main text. **d**, Activity of hPAD4 in the presence of control antibody, an inhibitory Fab to human PAD4 (hI281) or activating Fabs (hA288, and hA362) measured by citrullination of protein substrate H3. hI281 reduces the activity of PAD4 while hA288–hA362 increases PAD4 activity. **e**, Activity of mPAD4 in the presence of control antibody, an activating IgG to murine PAD4 (mA342) and its variant mA342-c4 measured by citrullination of protein substrate H3. Representative blots were reproduced three times before inclusion in text. GFP, green fluorescent protein. Source data

We carried out two phage selection campaigns with a synthetic antibody Fab-phage library either in the presence of 10 mM free calcium or EDTA to sample the active and inactive states of PAD4 as PAD4’s activity is dependent on calcium (Fig. 1a and Extended Data Fig. 1f)^1^. We observed that the thermostability of the Ca^2+^ bound state is dramatically higher than the apo-state, shown by a 20 °C upward shift in the melting temperature, demonstrating that the enzyme exhibits two distinct conformational states with or without calcium (Extended Data Fig. 1g,h). Four rounds of selections were performed with either 10 mM Ca^2+^ or 1 mM EDTA, respectively. To increase the stringency of selection, we systematically decreased the PAD4 concentration in every subsequent round of selection. At the end of the fourth round of selection, 95 individual clones were screened for binding by Fab-phage enzyme-linked immunosorbent assays (ELISAs). The top ten binders from both the Ca^2+^ and EDTA selections were sequenced and expressed. We then performed a second hPAD4 selection to identify PAD4 activators and inhibitors that targeted different epitopes from the ones discovered in our first selection campaign. To do this, we added hI281 in excess during selection to block this previously identified epitope. This strategy allowed us to discover new binders and create a toolkit of diverse PAD4–antibody modulators. The top binding clones from our second selection Fab-phage ELISAs were sequenced, affording 16 new anti-hPAD4 clones.

**Extended Data Fig. 2:**
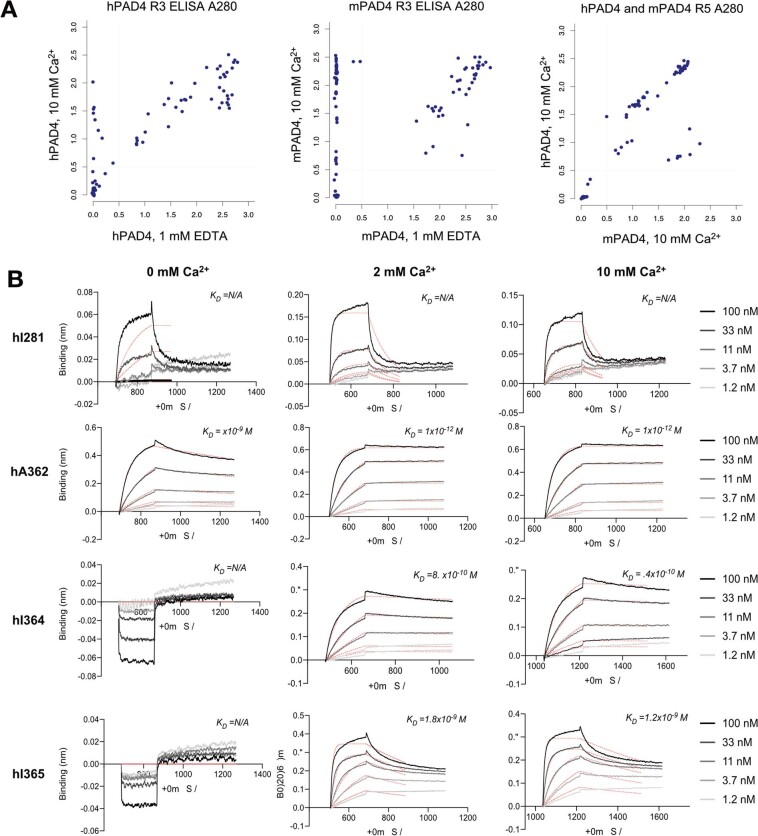
Phage ELISA for characterizing hPAD4/mPAD4 cross reactivity and multi-point biolayer interferometry (BLI) binding data of lead candidate IgGs to hPAD4. (**a**) Binding of Fab-phage to hPAD4/mPAD4 measured by ELISA A280 signal. Very few clones from hPAD4 or mPAD4 phage selections (left, middle) were cross reactive. By taking the round 3 phage pool and performing selections with the other antigen for two additional rounds, nearly all phage clones from round 5 are cross reactive with hPAD4 and mPAD4 (right). (**b**) Multi point BLI data show that hI364 and hI365 are dependent on calcium to bind hPAD4. hI281 and hA362 are calcium-independent binders although binding to the calcium-bound form of the enzyme is stronger. Binding of all clones to PAD4 at 2 mM Ca^2+^ is comparable to their binding at 10 mM Ca^2+^. K_D_s reported as <1 nM are due to bottoming out of off-rate measurements on BLI. Source data

We were also interested in identifying antibodies against mouse PAD4, as these binders can be used as tools for studying the role of PAD4 in mouse models of RA and mimic the development of anti-PAD4 autoantibodies in patients with RA^28^. We carried out an additional phage selection campaign against mPAD4 and after four rounds of selection, 12 antibodies that exclusively bind to mPAD4 and 16 antibodies that were hPAD4–mPAD4 cross-reactive were identified (Extended Data Fig. 2a).

### Characterization of PAD4 antibodies

We used two established PAD4 activity assays for initial characterization of the functional consequences of antibody binding. The first is an end-point immunoblot assay that detects citrullination of a large natural protein substrate, histone H3. The second is a spectrophotometric assay using a small-molecule trypsin-fluorogenic substrate pair in which the substrate citrullination by PAD4 prevents hydrolysis by trypsin, so that a reduced fluorescence readout is proportional to higher PAD4 activity (Fig. 1b,c)^29^. As expected, PAD4 activity increases with higher concentrations of Ca^2+^ as measured by each assay. We used the more rapid and higher-throughput trypsin and/or fluorogenic peptide assay to screen the function of antibodies identified through our phage selection strategy.

The naming of our antibodies was as follows: the first letter (h or m) stands for human or mouse PAD4 to which the antibody binds. If the antibody is cross-reactive to human and mouse, we designate it as hm. The second letter (I, A or N) means inhibitory, activating or neutral, respectively. The letters are followed by the plasmid ID number. We found inhibitory, activating and neutral binders toward both hPAD4 and mPAD4 and summarize their binding and functional properties in Supplementary Table 1.

We identified five antibodies to hPAD4 (hI281, hA288, hA362, hI364 and hI365) that showed either antagonistic or agonistic effects (Fig. 1c). hI281 and hA288 were identified as the strongest inhibitors and activators from the first selection, respectively, and these binders were then used in a second selection against hPAD4 to mask their epitopes for exploring other binding pockets. This epitope blocking strategy allowed us to identify activating antibody hA362, and inhibitory antibodies hI364 and hI365 that bind to different epitopes from hI281 and hA288 (Fig. 1c). Our PAD4 modulating binders from the second selection also inhibited or activated PAD4-mediated histone H3 citrullination, indicating that these PAD4 binders can modulate citrullination of protein substrates as well as small synthetic substrates (Fig. 1d,e).

Some of our most potent inhibitors (hI364, hI365) exhibited selective binding to the Ca^2+^-bound state of PAD4 and showed either weak or no binding to the apo-enzyme in the presence or absence of Ca^2+^ as measured by biolayer interferometry (BLI) (Extended Data Fig. 2b). These results indicate that there are epitopes formed in only the Ca^2+^ bound form of PAD4 that are critical to enzyme activity. We believe that our calcium-dependent antibodies are targeting these critical epitopes, thus inhibiting activity.

**Extended Data Fig. 3:**
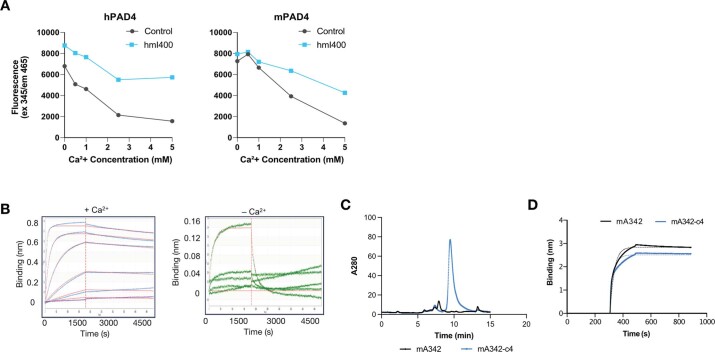
Functional characterization of mPAD4 and h/mPAD4 cross reactive antibodies. (**a**) hmI400 was identified as a functional inhibitor of both hPAD4 and mPAD4. (**b**) BLI results show mA342 interacts with the calcium bound form of mPAD4 but binds minimally to the calcium-free form. (**c**) SEC trace showing improved biophysical properties of mA342-c4 as compared to WT mA342. (**d**) BLI comparison of WT mA342 and its improved variant mA342-c4 in 10 mM Ca^2+^. Source data

Our top binders were also tested for cross-reactivity between mPAD4 and hPAD4 as this would be a useful feature for studying PAD4 between mouse and human. However, most binders identified from single species selections lacked cross-reactivity when terminally evolved for five rounds. This may be attributed to the fact that hPAD4 and mPAD4 share only 73% sequence homology and contain notable amino acid differences near important structural regions. For example, the N-terminal domain calcium-binding pocket contains several notable mutations from human to mouse PAD4 (D157E, E170K and D388N). We hypothesize that these changes may prevent the identification of cross-reactive binders to this important of region of enzyme regulation. In a further effort to identify pan-binding clones we took the eluted phage pool from round three of selection on mPAD4, and continued to select an additional two rounds on hPAD4. This selection on dual antigens greatly enriched for cross-reactive binders and we found one clone (hmI400) to inhibit both hPAD4 and mPAD4 (Extended Data Fig. 3a).

### Binding of antibodies to human and mouse PAD4

We characterized the binding affinity of several inhibiting and activating antibodies to human or mouse PAD4 using BLI. The binding affinity of each antibody was investigated under varying concentrations of calcium (0, 2, 10 mM). Of our inhibitory lead clones, hI364 and hI365 both exhibited no binding to PAD4 in calcium-free conditions but showed high affinity to PAD4 in 2 and 10 mM Ca^2+^ (for hI364 the dissociation constant *K*_d_ = 0.64 nM; for hI365 *K*_d_ = 1.2 nM). The binding of other clones (hI281, hA362) remained unaffected by calcium concentration (Extended Data Fig. 2b).

We similarly characterized the binding of our mPAD4 binders and identified mA342 to be a potent activator of mPAD4. This clone also exhibited calcium selectivity as its *K*_d_ improves from 200 μM to less than 1 nM on addition of 10 mM calcium (Extended Data Fig. 3b). However, mA342 tended to aggregate in solution and suffered from poor biophysical properties characterized by a low intensity, widely dispersed peak on size-exclusion chromatography (SEC) (Extended Data Fig. 3c). We hypothesized that the poor solubility was due to several hydrophobic residues in the complementarity determining regions (CDRs). To test this, we performed an alanine scan of the CDRs to identify hotspots of hydrophobicity to improve the solubility of mA342.

The alanine scan identified four hydrophobic CDR residues that were dispensable as they did not affect binding of mA342 to mPAD4. These four residues (light chain I97; heavy chain Y101, Y111, M114) were mutated to alanine to generate mA342-c3, which showed a clean, monodispersed peak on SEC, and improved binding and activation of mPAD4 measured by BLI (Extended Data Fig. 3c,d). We believe that mA342-c3 could be a promising candidate for mouse models mimicking the pathology of activating anti-PAD4 autoantibodies.

### Several antibodies modulate hPAD4 enzyme dimerization

**Fig. 2:**
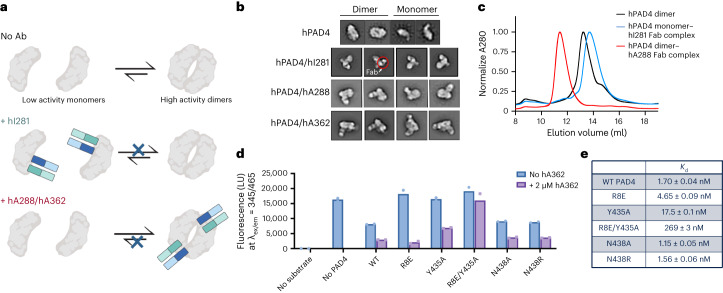
Antibodies that modulate hPAD4 dimerization influence enzymatic activity. **a**, Summary diagram of antibodies influencing PAD4 activity through modulating its dimerization. Monomeric PAD4 is less active than dimeric PAD4, and hI281 blocks dimerization while hA288 and hA362 promote dimerization. **b**, NSEM 2D micrographs showing monomeric state of PAD4 in complex with hI281 and dimeric state in complex with hA288 or hA362. **c**, SEC traces of PAD4 alone, in complex with Fab-hI281 and in complex with Fab hA362. PAD4 alone exists in both the monomer and dimer form, while addition of hI281 promotes the monomer form and hA362 promotes the dimer form as evidenced by elution time. **d**, Binding is correlated with antibody function using a trypsin substrate assay. Biological duplicates are shown. Fabs binding to mutants with less affinity are less able to inhibit enzymatic activity (right). **e**, Table displaying measured binding affinities of activating Fab hA362 to PAD4 mutants (left). Binding is drastically decreased on mutation of several residues at the PAD4 dimer interface. Source data

We used negative-stain EM (NSEM) to screen the overall shapes of PAD4 and the PAD4–antibody complexes that are schematically represented in Fig. 2a. As expected from X-ray structures of PAD4, the enzyme formed a C-shaped monomeric species and a globular, dimeric species of hPAD4 (Fig. 2b). Previous reports have shown that PAD4 exists in both low activity monomers and highly active dimeric states^30^. We next incubated hPAD4 with our functional antibody Fab fragments in equimolar amounts and characterized the stoichiometry of the protein complexes by NSEM. The antibody Fabs showed the typical donut-shaped structures in the EM micrographs. A 1–1 complex of hPAD4 to antibody was seen for hPAD4 in complex with the inhibitory antibody, hI281, while a 2–2 complex of hPAD4 to antibody was observed for hPAD4 in complex with both activating antibodies, hA288 and hA362 (Fig. 2b). Taken together, these data suggest that the inhibitor (hI281) may be blocking PAD4 dimerization as the antibody is only seen interacting with monomeric PAD4, while activators (hA288 and hA362) are promoting PAD4 dimerization and promoting activity.

We confirmed the stoichiometries for 1/1 inhibitor/PAD4 (120 kDa) and 2/2 activator/PAD4 (240 kDa) complexes by analytical SEC (Fig. 2c). The free hPAD4 shows a major dimeric peak and a minor monomeric peak on SEC reflecting its dimer to monomer equilibrium. The hPAD4–hA288 complex runs as a homogeneous species at its expected molecular weights of a 2–2 complex, whereas the hPAD4–hI281 complex runs at the molecular weight of a 1–1 complex. These data confirm that hI281 stabilizes the hPAD4 monomer, forming a 1–1 complex with hPAD4, while hA288 promotes hPAD4 dimerization to a 2–2 complex.

### PAD4 dimerization influences activity and antibody function

**Extended Data Fig. 4:**
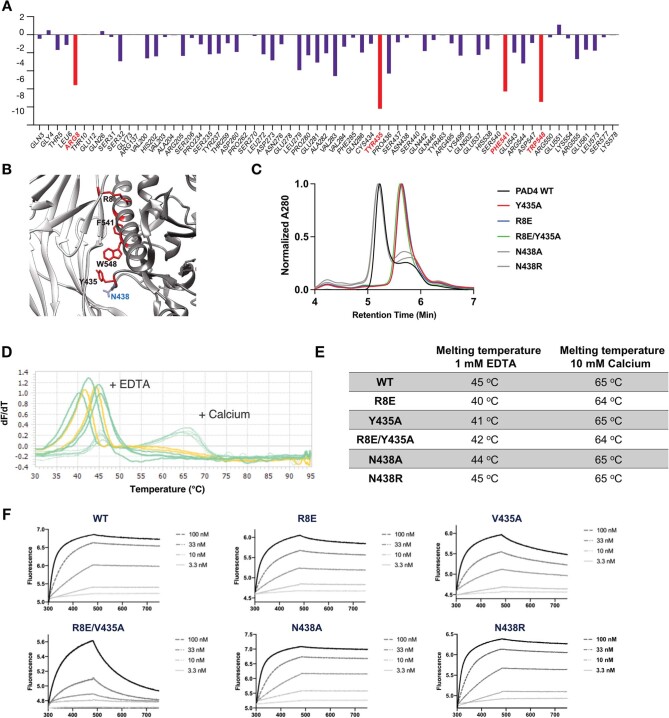
Biophysical characterization of PAD4 monomerization mutants and effect of PAD4 monomerization on antibody binding. (**a**) Computational modeling of the PAD4 dimer interface to identify key residues that contribute to dimerization (highlighted in red). (**b**) Key residues R8, Y435, and W548 highlighted at the dimer interface in red. The selected control residue N438 is highlighted in blue. (**c**) SEC trace showing earlier elution of dimeric, WT PAD4 compared to monomerization-promoting mutants R8E and Y435A. Control mutants, N438A and N438R (gray), elute with WT PAD4. (**d**). DSF plot of PAD4 mutants in the presence of 10 mM Ca^2+^ (green) or 1 mM EDTA (yellow). (**e**) Estimated T_m_s of PAD4 mutants from DSF plots. (**f**) BLI measurements of hA362 binding to WT hPAD4 and monomerization mutants. Source data

To further study how dimerization influences enzyme activity, we applied computation-guided mutational analysis to disrupt and test the PAD4 dimerization interface. Briefly, after relaxing and minimizing the structures in Rosetta (Protein Data Bank (PDB) 1WDA), we used PyMOL to select interacting residues within 5 Å from the two subunits. The interface energy function in Rosetta was applied to calculate an interaction score of different residues. Residues R8, Y435, F541 and W548 stood out as the top four residues contributing to interface association with large negative interaction scores (Extended Data Fig. 4a). We chose R8 and Y435 located at the two ends of the interface for a mutational analysis (Extended Data Fig. 4b). The interface energies of single mutant R8E, Y435A and double mutant R8E–Y435A were calculated to be notably higher than the wild-type (WT), indicating these mutations would be effective in breaking dimerization. R8 and Y435 were also reported in a previous mutational study to influence the PAD4 dimerization constant^30^. Additionally, PAD1 is the only PAD isoform that exists as a monomer in solution. This finding is likely explained by an R8Q substitution in the N-terminal domain of PAD1, again supporting the importance of R8 in enzyme dimerization^31^.

Based on these analyses, we cloned and expressed the R8E, Y435A and double mutant R8E–Y435A of PAD4. We also expressed two additional mutants as negative controls, N438A and N438R, as we predicted residue N438 to minimally contribute to the interface energy. Indeed, we found that R8E, Y435A and R8E–Y435A ran as monomers on SEC while the WT, N438A and N438R form dimers (Extended Data Fig. 4c). All monomeric mutants were virtually inactive in the peptide citrullination assay while N438A and N438R had comparable activities to WT hPAD4 (Fig. 2d). These data confirmed that dimerization is important for PAD4 activity^19,30^. We also found the monomerizing mutations slightly destabilized the enzyme, reducing the melting temperature of apo-PAD4 from 45 to 40–42 °C, and Ca^2+^-bound PAD4 from 65 to 64 °C (Extended Data Fig. 4d,e).

We next studied how WT and mutant PAD4s bind to the activating antibody hA362 that promotes enzyme dimerization. The binding affinity of hA362 was decreased by about three-, ten- and 160-fold for R8E, Y435A and the R8E/Y435 double mutant, respectively, and we noticed that their ability to activate PAD4 decreased in rough proportion to their reduction in binding affinity. While the Y435A mutation directly affects the hA362 binding epitope, the R8E mutation alters a series of electrostatic interactions that also exist near the epitope, thus explaining the observed reduction in binding affinity and antibody functionality. By contrast, the null mutants, N438A and N438R, did not affect antibody binding (Fig. 2e and Extended Data Fig. 4f). These results further support that the binding and activation efficacy of hA362 is influenced by dimerization of PAD4.

### hA362 activates hPAD4 by stabilizing dimerization

**Fig. 3:**
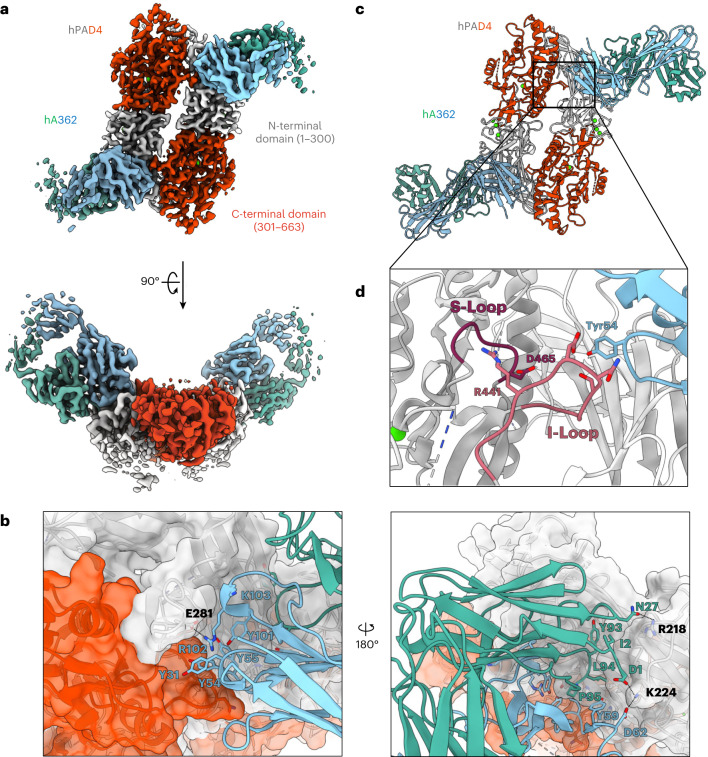
hA362 directly contributes to the PAD4 dimerization interface and helps order the substrate-binding site. **a**, Two views of the cryo-EM map of PAD4 in complex with Fab hA362 (PDB 8SMK). PAD4 monomers are in shades of gray (N-terminal domain) and red (C-terminal domain), hA362 heavy and light chains are teal and blue. **b**, Model of PAD4–hA362 derived from the cryo-EM map shown as ribbon. Boxed region delineates Fab CDR interaction with the I- and S-loop on PAD4. **c**, Zoomed-in view of the boxed region in **b** shows hA362 reaching across the PAD4 monomer to interact with the I-loop on the other PAD4 monomer. This helps order the S-loop via the R441-D465 salt bridge. **d**, Detailed hA362–PAD4 interactions. Both chains of the Fab pack a large number of aromatics against both monomers of PAD4 dimer. Ion and hydrogen bonds marked with dashed lines.

**Extended Data Fig. 5:**
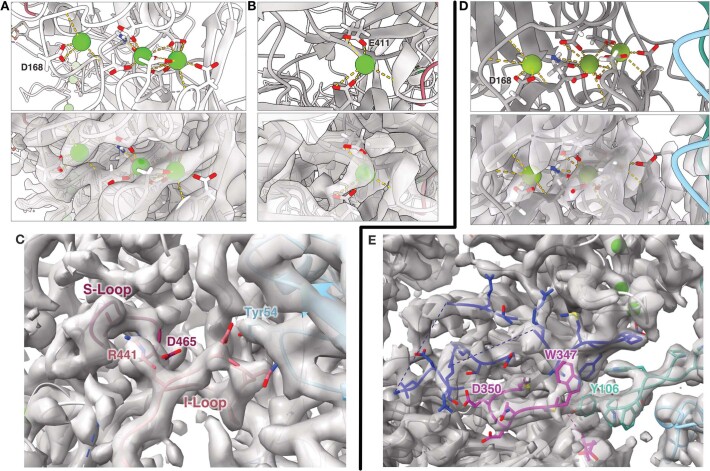
Detailed view of Ca^2+^ ions and Fab/hPAD4 interactions with cryo-EM map. (**a**) Three Ca^2+^ ions in the N terminal domain of hPAD4-hA362 structure. Model in ribbon and atoms on top, overlayed with cryo-EM map on the bottom. (**b**) Ca^2+^ ion in the C terminal domain of the hPAD4-hA362 structure. One Ca^2+^ was removed from analysis as the electron density was very weak. (**c**) Zoomed in view of the hA362 interactions with the hPAD4 I-loop with corresponding cryo-EM map. Adapted from Fig. 3c. (**d**) Ca^2+^ ions in the N terminal domain of the hPAD4-hA365 structure. (**e**) Zoomed in view of the hI365 interactions with the hPAD4 active site with corresponding cryo-EM map. Adapted from Fig. 4c**, right panel**.

To further understand the molecular mechanism of hA362 activation of PAD4 we obtained the cryo-EM structure of hPAD4 in complex with hA362 at 3.5 Å resolution. The overall structure is a 2–2 complex, containing two copies of hA362 bound to a homodimeric PAD4 (Fig. 3). Consistent with previous structures of the enzyme alone, PAD4 forms an anti-parallel head-to-tail homodimer in C2 symmetry^32^. Each PAD4 monomer adopts an elongated fold with a N-terminal immunoglobulin-like structure and a C-terminal α/β propeller structure, and each monomer binds to a total of five Ca^2+^ ions in two distinct pockets (Extended Data Fig. 5a,b).

Although each hA362 Fab predominantly interacts with the N-terminal domain of each PAD4 (buried surface area 1,196.3 Å^2^), the Fab heavy chain (HC) spans across one PAD4 monomer to make an additional interaction with the C-terminal domain of a second PAD4, close to the substrate-binding site (buried surface area 183.9 Å^2^) (Fig. 3b). This readily explains why the binding affinity of hA362 is lower to the monomeric PAD4 mutants as monomerization eliminates a portion of the binding epitope. This additional contact with the PAD4 in *trans* is with the interface loop (the I-loop, shown in red), a known regulatory motif (Fig. 3c,d and Extended Data Fig. 5c). The specific interaction of hA362 with the I-loop mechanistically explains why PAD4 activity is enhanced, as the substrate-binding loop (S-loop, shown in purple) neighbors the I-loop. The I-loop engages the S-loop through an electrostatic interaction between R441 and D465 and this interaction is important for catalytic activity^32^. Consistently, previous molecular dynamics simulations have found that the I-loop in a monomeric PAD4 W548 mutants exhibits high flexibility, accounting for the increased flexibility of the S-loop and low activity of monomeric PAD4 (ref. ^32^). Our structural results further show that the I-loop and the associated S-loop can be stabilized through hA362 binding and directly facilitating organization of the active site.

### hPAD4 inhibition by re-structuring binding pockets

**Fig. 4:**
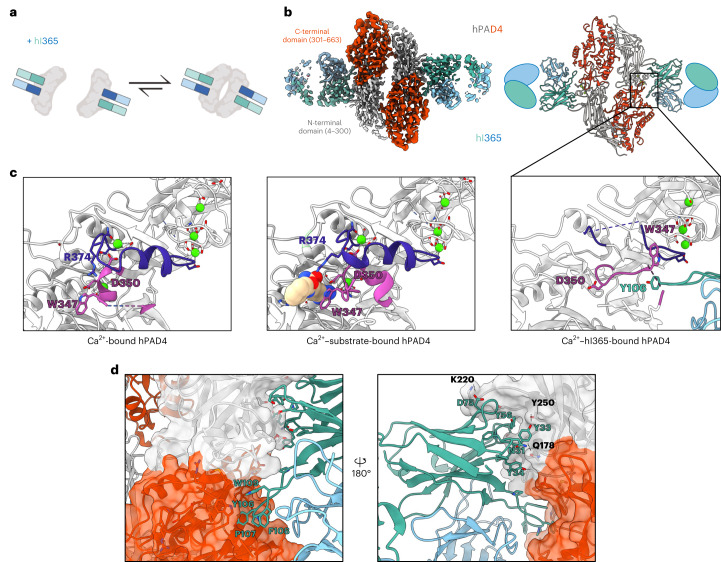
Cryo-EM structure illustrates the mechanism of calcium dependency and inhibitory function of hI365. **a**, Schematic showing that inhibitory Fab hI365 is able to bind both the monomeric and dimeric form of hPAD4. **b**, Cryo-EM map (left, PDB 8SML) and the resulting model (right) of hPAD4 (N-terminal domain, gray; C-terminal domain, red) in complex with hI365 (HC, teal; LC, blue). The Ab primarily binds an N-terminal Ca^2+^-coordinated region, but the H3 loop extends to the C-terminal pocket and induces conformational change. **c**, Comparison of the Ca^2+^-bound hPAD4 (PDB 1WD9), Ca^2+^–substrate bound hPAD4 (PDB 1WDA), and the Ca^2+^–hI365 bound hPAD4. The interaction of hI365 with residues 340–352 in hPAD4 (magenta) alters the structure and orientation of this fragment disrupting the organization of several residues (D350, R374, W347) involved in calcium and substrate binding. **d**, Detailed hA365–PAD4 interactions. The Fab interacts predominantly with one chain against one monomer of PAD4, burying a considerable number of aromatics with some hydrogen bonds.

Fabs hI364 and hI365 are two of the most potent inhibitors we identified through our selection. Both Fabs only recognize the Ca^2+^-bound form of PAD4. We determined the cryo-EM structure of hPAD4 in complex with hI365 at 3.2 Å resolution to understand the antibody calcium dependency and inhibitory mechanism (Fig. 4b). Similar to hA362, hPAD4 adopts a homodimeric structure and forms a 2–2 complex with hI365 (Fig. 4a,b), but here each antibody only directly interacts with one PAD4 monomer (buried surface area 1,122.9 Å^2^). The CDRH1 and H2 of hI365 binds to a region that is structured only upon binding of three Ca^2+^ ions in the N-terminal domain, explaining why the binding of hI365 is Ca^2+^-dependent (Fig. 4b).

Each hPAD4 monomer in the hI365–hPAD4 complex only contains three of the five Ca^2+^ ions that are typically bound in PAD4 structures and the hA362–PAD4 complex (Extended Data Fig. 5d). A closer look of the structure reveals that binding of hI365 to hPAD4 leads to the occlusion of both Ca^2+^ ions in C-terminal domain while the ions in the N-terminal domain remain bound (Fig. 4c and Extended Data Fig. 5e). Additionally, the Y106 residue in CDR H3 loop of hI365 interacts with Trp347 in hPAD4 (Fig. 4c, pink), pulling the 340–352 loop out of the substrate-binding site and disrupting a small helix usually formed in the active structures (residues 374–383)^33^. In the original hPAD4 structure (PDB 1WDA) with substrate benzoyl-l-arginine amide, the 340–352 loop contains key residues for interactions with both the C-terminal Ca^2+^ ions and the hPAD4 substrate^33^. The 347–350 region also stabilizes R374, which binds substrate, along with the surrounding Ca^2+^ binding region (purple). R374 and the nearby helical region are also no longer resolved with hI365 binding (Fig. 4c, right panel and Extended Data Fig. 5e). Therefore, structural alteration of these residues diminishes the ability of the protein to bind calcium in its C-terminal domain and the substrate in this pocket. The negatively charged D350 residue is known to stabilize binding of the arginine substrate, and mutation of D350 results in loss of enzyme activity^33^. Our structure shows that binding of hI365 to PAD4 results in D350 flipping away from the substrate pocket, thus leading to enzyme inhibition (Fig. 4c). Additionally, hI365 forms many hydrophobic interactions with one predominant PAD4 monomer although loop H3 also interacts with the second PAD4 monomer (Fig. 4d). These results reveal another mechanism for blocking PAD4 activity, namely pulling out loop 340–352 and preventing Ca^2+^ and substrate from binding.

### Optimized hI365 shows potent inhibition of hPAD4 in vitro

**Fig. 5:**
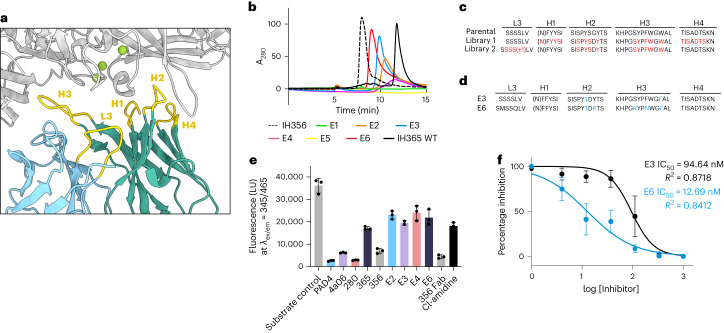
Antibody engineering strategies used to improve affinity and inhibition activity of the calcium-dependent binder, hI365. **a**, hPAD4–hI365 binding interface with CDRs L3, H1–H4 highlighted. CDR loops H1–H4 are forming contacts with PAD4, but L3 is too short to facilitate any contact with the enzyme. **b**, SEC traces of WT hI365 and affinity matured clones. Selected hits show improved SEC profiles as indicated by earlier elution times. A280, absorbance at 280 nm. **c**, CDR sequences of WT hI365 aligned with residues to be mutagenized via soft randomization; L3 is randomized to contain nine or ten amino acids. **d**, CDR sequences of the two top engineered binders with good affinity and solubility. **e**, PAD4 activity as measured by the fluorescent-substrate assay shows that several Ab clones identified from soft randomization inhibit PAD4 more potently than WT hI365. 2 mM Ca^2+^ was used in this assay to mimic physiological extracellular conditions. Error bars represent mean ± s.d. of three biological replicates. **f**, IC_50_ of lead candidates E3 and E6 as measured by PAD4 citrullination of H3. IC_50_ of E3 and E6 determined to be 95 and 13 nM, respectively, and represent mean ± s.e.m. of three biological replicates. Source data

**Extended Data Fig. 6:**
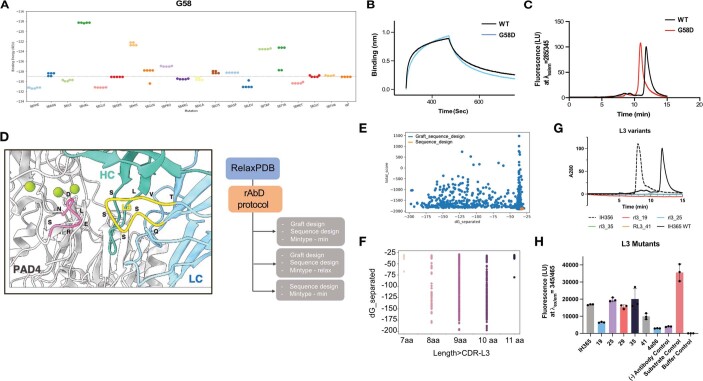
Rosetta antibody design (rAbD) guided optimization of hI365. (**a**) Several G58 mutants are predicted to reduce interface energy of hI365 and hPAD4. (**b**) BLI measurements showing that point mutant G58D binds PAD4 with similar affinity as WT hI365. (**c**) SEC trace showing improved biophysical properties of mutant G58D as compared to WT hI365, as evidenced by the earlier elution time. (**d**) Position of CDR L3 at the PAD4 binding interface and rAbD workflow for designing L3 variants. Though the loop sits at the interface, it is not forming any favorable interactions with PAD4 due to its short length. High resolution structure of PAD4/hI365 interface served as the input model for rAbD. CDR L3 of the relaxed model was allowed to ‘Graft’ and ‘Sequence’ design or only ‘Sequence’ design with or without flexible backbone design. Neighboring CDRs L1 and H3 were repacked to avoid clashes and optimize interface interactions. (**e**) ‘Graft’ and ‘Sequence’ design generated models (blue) with low totalscore and interface energies shown by dG_separated (in REU). ‘Sequence’ design alone did not improve the interface with all models (orange) having poor dG_separated score. The dG_separated score of the cryo_EM structure of PAD4/hI365 interface is ∼−54.4 REU (**f**) Rosetta antibody design (rAbD) predicted CDR L3 at lengths of 9 and 10 aa to have the most optimal interface energies. Different CDR L3 lengths and corresponding dG separated scores are shown. (**g**) SEC chromatagram showing poor performance of L3 mutants compared to parent (hI365) and control antibodies (hI356). (**h**) Fluorescent substrate activity assay showing inhibition of PAD4 in the presence of WT hI365 and the predicted L3 variants by rAbD. Error bars represent mean ± standard deviation of three biological replicates. Source data

While hI365 exhibited inhibitory properties against PAD4 in preliminary activity assays, the antibody performed inconsistently when attempting to obtain a half-maximum inhibitory concentration (IC_50_). In addition, we observed late elution of the antibodies on SEC, suggesting presence of sticky, hydrophobic residues that may contribute to assay variability by causing antibody aggregation (Fig. 5b). We thus used structural information to inform in silico and experimental methods to improve the solubility of hI365. First, using a Rosetta-based pairwise interaction analysis, we identified a G58D mutation that improved antibody solubility and SEC behavior (Extended Data Fig. 6a–c and Supplementary Note 2). Next we used the Rosetta antibody design algorithm to engineer light chain CDR loop 3 (L3). As seen in the cyro-EM structure, L3 is present at the binding interface but does not form any appreciable contacts to PAD4 (Fig. 5a and Extended Data Fig. 6d). We predicted that optimizing the length of L3, which is composed of nine amino acids in parental hI365, could promote additional contacts to PAD4. Computational screening of various randomized L3 grafts showed that the optimal length of L3 is nine to ten amino acids (Extended Data Fig. 6e–h). Though our L3 mutants bound PAD4 with similar affinity to WT hI365, all clones identified from our L3 engineering exhibited poor SEC profiles and did not approve the ability of the antibody to inhibit PAD4 (Extended Data Fig. 6g,h).

**Extended Data Fig. 7:**
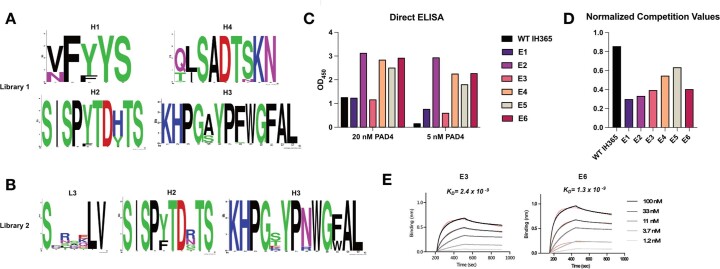
Soft randomization of hI365. (**a-b**) Sequence logos of library 1 and 2 showing amino acid preference for each CDR. (**c-d**) Binding of top Fab-phage clones to PAD4 as measured by direct and competition ELISAs. Top clones exhibit similar or improved binding to WT hI365 at both 20 nM and 5 nM PAD4. Top clones are less likely to be competed off PAD4, indicating a lower relative k_off_.(**e**) Multi-point octet trace of leading clones, E3 and E6. Both appear to bind PAD4 at nanomolar affinity. Source data

Last, a library-based randomization approach based on the knowledge gained by structure-informed in silico designs was carried out to simultaneously optimize binding and biophysical properties. We created two libraries. In library 1, all CDR H3 residues were targeted for soft randomization using a 70-10-10-10 formula^34^. In library 2, an RVK degenerate codon strategy was used at certain positions to bias mutagenesis to more hydrophilic residues (Fig. 5c). In both libraries, we incorporated information gained from our Rosetta antibody design algorithm engineering of CDR L3 and randomized this loop to contain both nine and ten amino acids. After four rounds of panning, we identified six unique clones that improved binding to hPAD4 compared to the parent hI365 (Extended Data Fig. 7a–d). Binding was initially measured via ELISA at 20 and 5 nM hPAD4 antigen, and results show that library-based clones outperformed the parental hI365, especially at 5 nM hPAD4 (Extended Data Fig. 7c). To estimate antibody off-rates, we preformed an Ab–hPAD4 complex before adding the mixture to hPAD4-coated ELISA plates. While hI365 dissociated from the initial Ab–PAD4 complex and readily bound plate-bound hPAD4 antigen, the newly identified library clones remained associated to hPAD4 in the preformed complex, suggesting an improvement in affinity (Extended Data Fig. 7d).

In particular, clones E3 and E6 showed dramatically improved SEC profiles, binding kinetics measured via BLI, and a marked increase in PAD4 inhibition compared to hI365 (IC_50_ values of 94 and 13 nM, respectively; Fig. 5d–f and Extended Data Fig. 7e). E3 and E6 both incorporate the G58D mutation previously shown to improve antibody solubility (Fig. 5d). In agreement with our computational CDR L3 studies, parental hI365 and E3 have a nine amino acid L3 loop length, while clone E6 has a ten amino acid long loop length. Thus, the affinity and the developability profiles of these antibodies were successfully improved by structure-informed antibody optimization, making them more suitable probes of PAD4 function.

### Antibody–PAD4 specificity and broader substrate profiling

**Extended Data Fig. 8:**
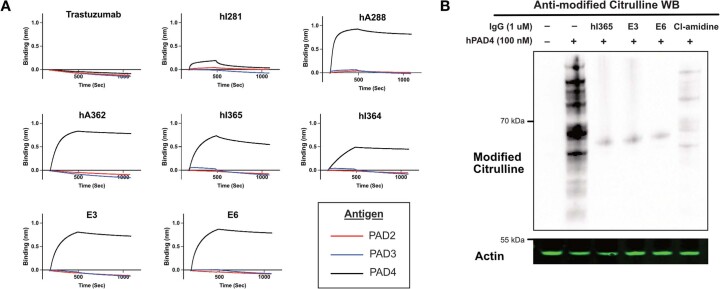
Anti-PAD4 antibody specificity and effect on PAD4 on whole cell lysate detect via anti-modified citrulline western blot. (**a**) Binding of hI365, E3, and E6 to PAD4, PAD2, PAD3, and Trastuzumab negative control measured by BLI. All PAD4 antibodies bind only to PAD4 and not PAD2 or PAD4. (**b**) Whole cell lysate treated with PAD4 +/- inhibitors. Functional antibodies, hI365, E3, and E6 inhibit PAD4-catalyzed citrullination on a variety of cytosolic, nuclear, and membrane-bound proteins. Trastuzumab IgG and pan-PAD inhibitor Cl-amidine included as negative and positive controls. An anti-actin loading control is included. Source data

While current small-molecule inhibitors show poor specificity for PAD4 over other PAD isoforms, we believe that our antibodies may offer the advantage of improved specificity that is commonly noted with biologics. We tested binding of our functional human antibodies (hI281, hA288, hA362, hI364, hI365, E3 and E6) against PAD4 alongside PAD2 and PAD3 via BLI, and all antibodies exhibited binding to PAD4 exclusively (Extended Data Fig. 8a).

In addition to developing PAD4-specific binders, we wanted to ensure that our antibodies inhibited PAD4 in biologically relevant environments. As extracellular PAD4 activity is hypothesized to be pathogenic in RA, we performed a PAD4 activity assay against whole cell lysate containing cytosolic, nuclear and membrane-bound proteins. While PAD4 alone citrullinated a variety of proteins present in whole cell lysate, our functional antibodies retained the ability to inhibit PAD4 (Extended Data Fig. 8b).

## Discussion

New therapeutic mechanisms for autoimmune diseases such as RA are in high demand as current approved treatments are limited in scope or susceptible to broad-spectrum resistance^35^. Upregulation of the expression and/or activity of PAD4, one of the five PAD isozymes expressed in the nucleus of bloodstream granulocytes, is associated with various autoimmune diseases including RA, Alzheimer’s disease, multiple sclerosis, lupus, Parkinson’s disease and cancer^36,37^. Genetic studies have also found an association between polymorphisms in PAD4 gene expression and RA disease risk, while PAD4 knockout or pharmacological inhibition through small molecules have proved to reduce disease severities in several RA mouse models^38^. These results have inspired a recent rise in interest for developing PAD4 inhibitors as a new generation of RA therapeutics.

Blocking PAD4-mediated extracellular citrullination is a promising strategy for autoimmune intervention, but further validation and mechanistic interrogation need to be performed and requires new tools^39^. Antibodies are established protein tools and have been commonly used to bind under specific conditions including pH and high calcium concentrations to target different protein conformations to interrogate their function^40,41^. Here, using an unbiased, Fab-phage display approach, we discovered conformation-selective antibodies and built a toolkit of both inhibitory and activating antibodies to influence PAD4 activity. We believe these function-modulating antibodies can aid the study of PAD4’s involvement in disease pathology and ultimately reveal insights for designing alternative and unique treatments of RA.

Our study reports structures of antibody binders in complex with PAD4, and our findings identify several allosteric mechanisms that may be harnessed for PAD4 activation or inhibition. The first mechanism is modulation of dimerization. Previous studies have discussed the impact of PAD4 oligomerization state on enzyme activity as structural changes along the dimerization interface heavily affect formation of the enzyme’s substrate-binding loop^3,29,31^. Although the monomeric form remains active, *k*_cat_ values of previously reported PAD4 monomerization mutants are decreased as much as fourfold compared to the WT enzyme. These results provide a rationale for developing PAD4 inhibitors that function as dimerization blockers. In our study, we identified several binders, hI281, hA288 and hA362, that affect PAD4 dimerization and PAD4 function. In particular, hI281 blocks enzyme dimerization. Blocking enzyme dimerization with hI281 effectively inhibits enzyme activity, just as PAD4 monomerization mutants are also less active. On the other hand, the activating antibodies, hA288 and hA362, promoted dimerization and catalytic activity. Through cryo-EM analysis of the Fab–PAD4 complexes, hA362 was found to bind and stabilize the interface loop, which has previously been shown to stabilize the substrate-binding loop on PAD4 dimer formation^31^. These findings both reinforce previously known PAD4 regulation mechanisms afforded by enzyme oligomerization state and show that functional antibodies can be used to both activate and inhibit PAD4 activity through this mechanism.

Along with dimerization, calcium binding also plays an important role in PAD4 function. Ca^2+^ binding in the C-terminal domain is vital in structuring the enzyme catalytic pocket, while Ca^2+^ binding in the N-terminal domain plays an important role in proper protein folding^1^. As PAD4’s catalytic pocket is partially disordered in the absence of C-terminal Ca^2+^ ions, this acidic pocket presents an allosteric site that directly affects PAD4’s ability to catalyze citrullination. As determined through cryo-EM, our antibody hI365 binds to a structural fragment in the C-terminal Ca^2+^ pocket. Once hI365 engages PAD4, several key residues in this pocket are rearranged, inhibiting Ca^2+^ binding and subsequently leading to disruption of substrate binding and loss of enzymatic activity. hI365 also recognizes the folded Ca^2+^ site in the N-terminal domain, thus exhibiting selective binding to the PAD4 only in the presence of Ca^2+^. Specifically targeting calcium-bound PAD4 presents an intriguing strategy for RA treatment as a calcium-form selective binder will ignore inactive, apo-PAD4. This could hypothetically improve the therapeutic index of an anti-PAD4 drug, as we would only target protein actively contributing to disease pathogenesis in the joints.

Additionally, our functional antibodies show high specificity for PAD4 over other PAD isoforms. Of the four other PAD isoforms (−1, −2, −3 and −6) we thought it was most relevant to test binding of our antibodies against PAD2 and PAD3, both of which share notable structural similarities to PAD4 (refs. ^42,43^). PAD2 is also implicated in various autoimmune diseases and ubiquitously expressed across all tissue, so there is a need for PAD4-specific tools to address its role separately from PAD2 in disease pathology. As for PAD3, it is known that a subset of patients with RA have PAD3 and PAD4 cross-reactive autoantibodies^22,23^. Given the precedence for PAD3 and PAD4–antibody cross-reactivity, we thought it would be important to show that our antibodies are still PAD4 specific. To address this, we tested the binding of our antibodies against commercially available PAD2 and PAD3 via BLI and found that none of our antibodies exhibit binding to PAD2 or PAD3. These PAD4-specific antibodies should be useful for isolating the functional effects of extracellular PAD4 from PAD2/3.

Taken together, we discovered functional antibody modulators of PAD4 through unbiased phage selection methods. These antibodies were key to reveal alternative mechanisms, direct and allosteric, for both PAD4 activation and inhibition. In the future, these highly specific and functional PAD4 binders may be used to investigate PAD4 activity in mouse arthritis models and human samples to shed light on how PAD4-mediated citrullination affects RA disease progression. Given the increasing evidence that PAD4 is a feasible anti-RA therapeutic target, using biologics to target extracellular PAD4 may provide a safer and more potent alternative for RA treatment.

## Methods

### Vector design and construction

We used a previously described vector for expression of Fabs in *E. coli*^44^. The pFUSE-hIgG1-Fc (InvivoGen) vector was used for expression of IgGs wherein the heavy chain was genetically fused to the hIgG1-Fc and the light chain was expressed on a separate copy of the vector without Fc. The vector used to express hPAD4 and mPAD4 was generated by Gibson cloning into the same vector for Fab expression. Each PAD4 was fused to an N-terminal His_6_-AviTag-PreScission or tobacco etch virus (TEV) cleavage site.

### Expression and purification of PAD4

C43 (DE3) Pro+ or BL21 Gold (DE3) *E. coli* containing PAD4 expression vectors were grown in 2xYT at 37 °C to an optical density at 600 nm of 0.4–0.8 and then protein expression was induced by the addition of 0.5–1.0 mM isopropyl-β-d-thiogalactoside. Incubation temperature was subsequently reduced to 18 °C and the cultures were allowed to shake for 16–20 h. Cells were gathered by centrifugation and lysed using sonication. The lysate was centrifuged to remove inclusion bodies. The enzymes were purified by Ni-NTA resin with 0.5 mM TCEP supplemented to all buffers to prevent PAD4 oxidation. The purified enzyme was buffer exchanged to 50 mM Tris (pH 8), 400 mM sodium chloride and supplemented with 0.5 mM TCEP. Purification steps were performed on ice to maintain high PAD4 enzymatic activity. Purified enzyme was aliquoted and flash frozen.

### Phage display selections

All phage selections were done according to previously established protocols. Briefly, selections with antibody phage library E were performed using biotinylated antigens captured with streptavidin-coated magnetic beads (Promega)^44^. Before each selection, the phage pool was incubated with streptavidin beads to deplete the library of any binders to the beads or sticky antibodies. In total, 3–5 rounds of selection were performed with decreasing amounts of PAD4 antigens (100, 50, 10, 10, 10 nM). Then 10 mM CaCl_2_ or 1 mM EDTA was added for Ca^2+^ or calcium-free selection schemes, and 0.5 mM TCEP was used in all buffers to keep thiols reduced.

### Phage library-based affinity maturation

Kunkel mutagenesis was used to incorporate mutagenized oligo pools into CDR regions as previously described^45–47^. Briefly, each soft randomization library was first generated by producing single-stranded DNA (ssDNA) in *dut-/ung- E*. *coli* cells. Phosphorylated oligos were annealed to the ssDNA and subsequently amplified to generate covalently closed circular DNA that was electroporated into SS320 *E. coli* cells, grown up and infected with M13K07 helper phage to generate our Fab-phage library. Selections using this library were then performed against PAD4 as described previously.

### Expression of Fabs

Fabs were expressed as previously described^44^. Briefly, C43 (DE3) Pro+ *E. coli* containing expression plasmids were grown in terrific broth (TB) at 37 °C in an autoinduction media for 6 h and incubation temperature was subsequently reduced to 30 °C where the cultures were allowed to grow for an additional 16–18 h. Cells were gathered by centrifugation, lysed and Fabs were purified by Ni-NTA resin. Fab purity and integrity were assessed by SDS–PAGE. Fab sequences are provided in Supplementary Note 1.

### Expression of IgGs

Expi293 (Life Technologies) cells were transiently cotransfected with two pFUSE (InvivoGen) vectors harboring either the IgG heavy chain or the IgG light chain at a mass ratio of 1/1. The ExpiFectamine 293 transfection kit (Life Technologies) was used for transfections as per the manufacturer’s instructions. Cells were incubated for 5 days at 37 °C in an 8% CO_2_ environment before the supernatants were harvested by centrifugation. IgGs were purified by protein A affinity chromatography or Ni-NTA resin and assessed for quality and integrity by SDS–PAGE. IgG sequences are provided in Supplementary Note 1.

### Phage ELISAs

ELISAs were performed according to standard protocols. Briefly, 96-well Maxisorp plates were coated with NeutrAvidin (10 μg ml^−1^) overnight at 4 °C and subsequently blocked with bovine serum albumin (2% w/v) for 1 h at 20 °C. Next, 20 nM of biotinylated PAD4 was captured on the NeutrAvidin-coated wells for 30 min followed by the addition of phage supernatants diluted 1:5 in ELISA buffer (TBS, pH 7.4, 0.05% Tween-20, 0.2% bovine serum albumin) for 30 min. Then 10 mM calcium or 1 mM EDTA were supplemented to buffers to determine binding with either the calcium-bound form or the apo form of PAD4. The bound phage was then detected using a horseradish peroxidase (HRP)-conjugated anti-phage monoclonal antibody (GE Lifesciences, catalog no. 27-9421-01) and imaged on a Tecan i-control (v.3.4.3.0) plate reader. For competition ELISAs, diluted phage supernatants and PAD4 were incubated for 30 min at 20 °C before addition to the NeutrAvidin-coated plates.

### DSF antibody stability assay

Fab or IgG samples in PBS were mixed with Sypro Orange dye (20× stock) to make a final antibody concentration of 2, 4, 8 or 16 µM and a 4× dye concentration. Then 10 µl of each mixture was transferred to a BioRad 384-well PCR plate and covered by a quantitative PCR Sealing Tape. The assay was preformed over a temperature range of 25 to 95 °C with a temperature ramping rate of approximately 0.5 °C per 30 s, and fluorescence was detected using a Roche LC480 Light Cycler. Due to instrumentation constraints, the ramp rate is set based on the number of data points acquired per °C. Currently this is set at 20 acquisitions per °C equating to a ramp rate of 0.03 °C s^−1^.

### PAD4 293-Flp-in cells

To construct the PAD4 expressing HEK293 cell lines, Flp-In HEK293 (ThermoFisher) cells were cotransfected with the pOG44 vector (ThermoFisher) and a construct encoding PAD4, PAD4 D350A or PAD4 with a green fluorescent protein Dronpa tag in the pcDNA5/FRT Mammalian Expression vector (ThermoFisher). Cells expressing PAD4 constructs were selected for in DMEM supplemented with 10% FBS, 100 µg ml^−1^ zeocin and 100 µg ml^−1^ Hygromycin B (ThermoFisher). Protein expression was confirmed by fluorescence microscopy detection of the Dronpa tag or PAD4 activity assays. To prepare cell lysates for PAD4 activity assays, cells were lysed in RIPA cell lysis buffer (50 million cells per ml) and supplemented with a protease inhibitor cocktail (Sigma-Aldrich). After rotating at 4 °C for 15 min, cell lysates were sonicated and spun at 16,000*g* to remove cell debris.

### Fluorescence-based PAD4 activity assay

PAD4 activity in the absence or presence of antibodies were assessed in a fluorescence-based assay with a pro-fluorescence substrate analog^29^. One µM of PAD4 was mixed with various concentrations of antibodies and calcium at 4 °C for 45 min, followed by the addition of 25 µM substrate ZRcoum, an arginine mimetic that releases a fluorophore once cleaved by trypsin. The reaction was incubated at 37 °C for 110 min. Upon addition of excess trypsin and EDTA to the solution, the fluorophore was liberated from uncitrullinated ZRcoum, but remained quenched and unmodified when the substrate was citrullinated. The reaction was read on a Tecan i-control (v.3.4.3.0) fluorescence plate reader with an excitation wavelength of 345 nm and an emission wavelength of 465 nm.

### Citrullinated histone H3 PAD4 activity assay

PAD4 activity in the absence or presence of antibodies was also assessed using a citrullinated histone H3 western assay. Ten to 100 nM recombinant PAD4 was mixed with antibodies with various concentrations of calcium at 4 °C for 45 min, then incubated with 760 nM recombinant histone H3.1 (New England Biolabs). The reaction was incubated at 37 °C for 110 min, followed by western analysis using an anticitrullinated H3 primary antibody (Abcam Ab5103) and an anti-rabbit HRP secondary antibody. For the PAD4 293-Flp-in cell lysate assay, 10 µl of cell lysate was mixed with varying concentrations of antibodies and calcium in a final volume of 19 µl at 4 °C for 45 min. The reactions were then incubated at 37 °C for 110 min and subsequently analyzed by western blot using an anticitrullinated H3 antibody (Ab5103) and anti-rabbit HRP secondary antibody. Images were acquired in Image Lab (v.5.0) and processed with Image Studio Software (v.5.2). IC_50_ measurements were obtained with technical triplicates and quantified using Fiji^48^.

### Modified citrulline western blot assay

Lysate was harvested from Expi293T cells using a 1× RIPA + protease inhibitor solution. Antibody–PAD4 complexes were preformed at 4 °C for 45 min. The complexes were then incubated at 37 °C for 110 min and subsequently analyzed by western blot using an antimodified citrulline detection kit (EMD Millipore). Polyvinyl difluoride membrane was incubated with anticitrulline probe overnight, then blocked and stained with a primary antimodified citrulline antibody and secondary HRP linked anti-IgG antibody. HRP signal was detected using a BioRad ChemiDoc imager.

### SEC

SEC analysis was performed using an Agilent HPLC 1260 Infinity II LC system equipped with an AdvanceBio SEC column (300 Å, 2.7 μm, Agilent). Each analyte was injected at 10 μM and run with a constant mobile phase of TBS high salt buffer (50 mM Tris, pH 8, 400 mM NaCl) for 15 min. Fluorescence (excitation 285 nm, emission 340 nm) and absorbance were measured and analyzed with Agilent OpenLab CDS ChemStation software.

### BLI

BLI measurements were made using an Octet RED384 (ForteBio) instrument. Biotinylated PAD4 was immobilized on optically transparent streptavidin biosensors (ForteBio) and loaded until a 1 nm signal was achieved. After blocking with 10 μM biotin, purified binders in solution were used as the analyte. TBSTB was used for all buffers. Data were analyzed using the ForteBio Octet analysis software and kinetic parameters were determined using a 1:1 monovalent binding model (https://www.sartorius.com/en/products/protein-analysis/octet-bli-detection/octet-systems-software).

### Negative-stain transmission electron microscopy

Here, 2.5 μl of PAD4 samples at 40 μg ml^−1^ were applied to a glow-discharged Cu grid covered by continuous carbon film, then stained with 0.75% (w/v) uranyl formate^49^. A Tecnai T12 microscope (ThermoFisher FEI Company) operated at 120 kV was used to analyze negatively stained grids. Images were recorded using an UltraScan 4000 camera (Gatan) at normal magnification of ×52,000 corresponding to a pixel size of 2.21 Å on the specimen.

### Cryo-EM studies of PAD4 in complex with hA362 and hI365

#### hPAD4–hA362

Sample concentrations used for cryo-EM were between 2 and 3 μM in 50 mM Tris pH 8.0, 150 mM NaCl, 10 mM CaCl_2_, 0.5 mM TCEP. Grids were frozen at 7 s blot time, 5 °C, 95% humidity on a Vitrobot. Videos were collected on a Titan Krios at 300 kV and 0.835 Å per pixel, 74 e^−^ total dose. Then 2,214 images were collected untilted; 1,620 films were collected at 15° tilt; 1,048 were collected at 30° tilt. CTF (constant transfer function) was estimated using Patch CTF in cryoSPARC2 (ref. ^50^). After curation, 1,593,955 particles were template picked. Then 3D classification was performed in cryoSPARC2. One class at 3.41 Å resolution with 274,910 particles was chosen for further refinement. These particles underwent two rounds of ab initio reconstruction into two models. After this point, C2 symmetry was imposed. The resulting stack of 134,775 particles went into one round of ab initio (two classes) and one round of heterogeneous refinement (two classes). One class with 92,424 particles underwent homogeneous refinement to 3.5 Å. These particles and the corresponding map were imported into cisTEM and auto refined to two classes^51^. Four rounds of local refinement into two classes were performed at increasing starting resolutions (10, 7, 5, 4.5 Å), leading to a final local refinement where one class with 59.78% (55,251) of the particles at 3.3 Å was chosen. Generate3D was then used to generate half maps. Local and global resolution maps were generated in cryoSPARC2, and directional resolution was determined with the 3DFSC server^52^.

#### Model building

**Extended Data Fig. 9:**
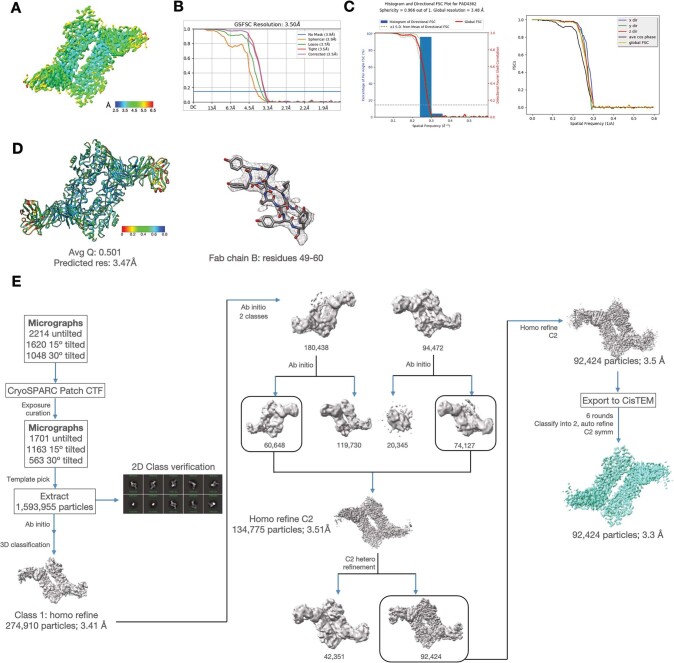
Cryo-EM map statistics for hPAD4/hA362. (**a**) Map colored by local resolution. (**b**) Global resolution FSC plots generated from half maps. Blue line indicates FSC = 0.143 cutoff. (**c**) Directional FSC plots. Left: Histogram showing the percentage of per angle FSC vs resolution, showing agreement with global FSC and sphericity of 0.966. Right: Directional FSC curves. (**d**) Left: Model colored by per residue Q score; Right: Fab CDR loop showing model/map fit. PDB: 8SMK. (**e**) Cryo-EM data processing workflow for PAD4-hA362.

PDB 1WD9 was fit, SWISS-MODEL was used with model Fab 6OTC (light chain) and 1N8Z (heavy chain) to build a homology model from the hA362 Fab sequence. This model then underwent Phenix Real-Space Refinement^53^, followed by Rosetta relaxation in torsion space. Loops that were not visible in the density were deleted with Coot. Isolde was then used for local adjustments to fix Ramachandran outliers and manual placement into the map density^54^. A final Rosetta torsion relax with C2 symmetry imposed was used to create the final model. Validation was performed in Phenix, and model-map correlation was performed with the MapQ plugin for UCSF Chimera^55,56^. Buried surface area was calculated with the PISA server (Extended Data Fig. 9).

#### hPAD4–hI365

Sample concentrations used for cryo-EM were between 2 and 3 μM in 50 mM Tris pH 8.0, 150 mM NaCl, 10 mM CaCl_2_, 0.5 mM TCEP. Grids were frozen at 7 s blot time, 5 °C, 95% humidity on a Vitrobot. As initial processing of untilited micrographs resulted in anisotropic resolution reconstruction, additional tilted data sets were collected. Videos were collected on a Titan Krios at 300 kV and 0.835 Å per pixel, 70 e^−^ total dose. 1,773 images were collected untilted; 1,844 videos were collected at 15° tilt and 842 were collected at 30° tilt. CTF was estimated with Patch CTF in cryoSPARC2. Bad micrographs were removed with the exposure curation tool, leading to 1,225 untilted, 1,366 at 15° tilt and 648 at 30° tilt. These underwent template picking, and 1,215,874 particles were picked. These underwent ab initio reconstruction into three classes; the best class with 780,324 particles was chosen. This class underwent ab initio reconstruction into two classes; the best class had 391,776 particles. These were re-extracted and particles were placed into five ab initio classes. Of these, 241 went through three classes of heterogeneous refinement; the best class with 143,413 particles at 6.3 Å was chosen. These particles then underwent two rounds of heterogeneous refinement with C2 symmetry imposed, leading to a stack of 73,648 particles, which were then imported into cisTEM for further processing. These were classified in two classes with Auto Refinement; both classes resulted in 3.9 Å maps. All particles then underwent four rounds of local refinement into two classes at increasing starting resolutions (8, 6, 5, 4.5 Å).

In the final round, class 1 was chosen with 62.22% (45,824) of the particles at 2.98 Å. Generate3D was then used to generate half maps. Local and global resolution maps were generated in cryoSPARC2, and directional resolution was determined with the 3DFSC server.

**Extended Data Fig. 10:**
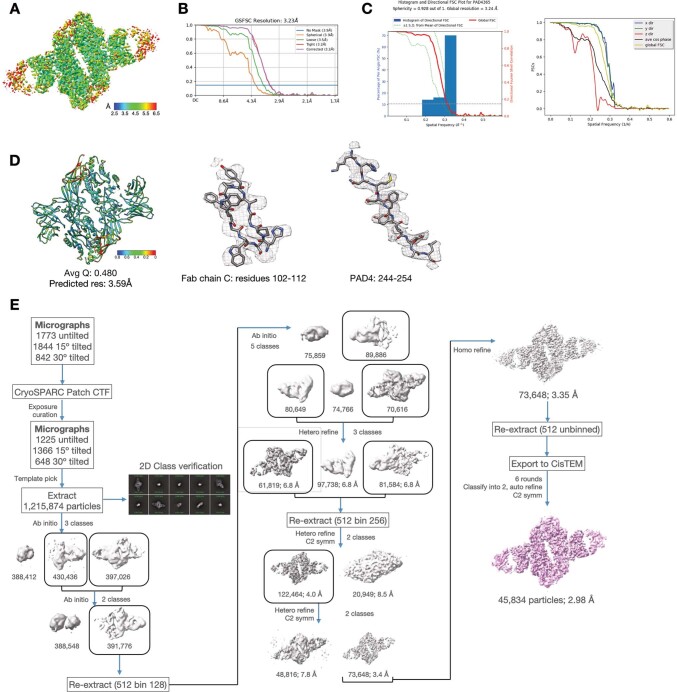
Cryo-EM map statistics for hPAD4/hI365. (**a**) Map colored by local resolution. (**b**) Global resolution FSC plots generated from half maps. Blue line indicates FSC = 0.143 cutoff. (**c**) Directional FSC plots. Left: Histogram showing the percentage of per angle FSC vs resolution, showing some degree of anisotropy, but overall agreement with the global FSC and sphericity of 0.928. Right: Directional FSC curves demonstrating better than 3 Å resolution in two directions and ∼4.5 Å in third direction. (**d**) Left: Model colored by per residue Q score; Right: Fab CDR loop showing model/map fit. PDB: 8SML. (**e**) Cryo-EM data processing workflow for PAD4-hI365.

For the model building, PDB 1WD9 was fit, SWISS-MODEL was used with model Fab 6OTC (light chain) and 1N8Z (heavy chain) to build a homology model with the 365 Fab sequence. Fab loops were deleted and RosettaES was used to rebuild the loops. This model was flexibly fit into the density with Rosetta torsion relax. Loops and domains that were not visible in the density were deleted with Coot. Manual placement of resolved regions that did not match the homology models into map density was performed in Isolde. Finally, Rosetta torsion relax was performed again with C2 symmetry imposed to produce a final model. Validation was performed in Phenix, and model-map correlation was performed with the MapQ plugin for UCSF Chimera. Buried surface area was calculated with the PISA server (Extended Data Fig. 10).

### Statistics and reproducibility

All representative SDS–PAGE protein gels and western blots shown were reproduced three times before inclusion in text.

### Reporting summary

Further information on research design is available in the Nature Portfolio Reporting Summary linked to this article.

## Online content

Any methods, additional references, Nature Portfolio reporting summaries, source data, extended data, supplementary information, acknowledgements, peer review information; details of author contributions and competing interests; and statements of data and code availability are available at 10.1038/s41589-023-01535-8.

### Supplementary information

Supplementary Information Supplementary Tables 1 and 2, antibody sequences and code.

Reporting Summary

### Source data

Source Data Fig. 1 Source data, unprocessed western blots.

Source Data Fig. 2 Source data.

Source Data Fig. 5 Source data, unprocessed western blots.

Source Data Extended Data Fig. 1 Source data, unprocessed SDS–PAGE gels.

Source Data Extended Data Fig. 2 Source data.

Source Data Extended Data Fig. 3 Source data.

Source Data Extended Data Fig. 4 Source data.

Source Data Extended Data Fig. 6 Source data.

Source Data Extended Data Fig. 7 Source data.

Source Data Extended Data Fig. 8 Source data, unprocessed western blots.

## Data Availability

Cryo-EM structural data are deposited in the Protein Data Bank and Electron Microscopy Data Bank. The hPADI4–hA362 complex is deposited as PDB 8SMK and EMD-40589. The hPADI4–hI365 complex is deposited as PDB 8SML and EMD-40590. The crystal structure of hPADI4 from PDB 1WDA was used for dimer interface modeling and for reference against our inhibitor-bound PAD4 cryo-EM complexes. Any additional information is available upon request. Source data are provided with this paper.
