# Supplementary material for: Antibody discovery identifies regulatory mechanisms of protein arginine deiminase 4
Source: Nat Chem Biol. 2024 Feb 2;20(6):742–50. doi: 10.1038/s41589-023-01535-8 (PMC11142921; doi:10.1038/s41589-023-01535-8)
Supplement: Supplementary file 1 — Supplementary Tables 1 and 2, antibody sequences and code. [file 41589_2023_1535_MOESM1_ESM.pdf]

nature chemical biology

Article

https://doi.org/10.1038/s41589-023-01535-8

Antibody discovery identifies regulatory mechanisms of protein arginine deiminase 4

In the format provided by the authors and unedited

Antibody	Target	Activity	Ca^{2+} dependency	Mechanism	Structural Analysis	Affinity (KD)	IC50	PDB ID
hI281	hPAD4	inhibitory	No	Promote monomerization	NSEM	-	-	-
hA288	hPAD4	activating	No	Promote dimerization	NSEM	-	-	-
hA362	hPAD4	activating	No	Promote dimerization	NSEM/ Cryo-EM	< 1 nM	-	PDB: 8SMK
hI364	hPAD4	inhibitory	Yes	-	-	< 1 nM	-	-
hI365	hPAD4	inhibitory	Yes	Disrupt Ca^{2+} and substrate binding	Cryo-EM	1.2 nM	Varied	PDB: 8SML
hI365-E3	hPAD4	inhibitory	Yes	Disrupt Ca^{2+} and substrate binding	-	2.3 nM	95 nM	-
hI365-E6	hPAD4	inhibitory	Yes	Disrupt Ca^{2+} and substrate binding	-	1.3 nM	13 nM	-
mA342	mPAD4	activating	Yes	-	-	< 1 nM	-	-
mA342-c4	mPAD4	activating	Yes	-	-	< 1 nM	-	-

Supplementary Table 1. Summary table of all anti-human/mouse PAD4 antibodies detailing binding affinity, functional properties, mechanism of action, and associated PDB IDs.

	PAD4/hA362	PAD4/hI365
**Data collection**		
Grids	Quantifoil R1.2/1.3 Au 300 mesh	
Vitrification method	FEI Vitrobot	FEI Vitrobot
Microscope	Titan Krios	Titan Krios
Magnification	105,000x	105,000x
Voltage (kV)	300	300
Stage tilt (°)	0, (15, 30)	0, (15, 30)
Detector	K3	K3
Recording mode	Counting	Counting
Dose rate (e−/pix/sec)	8, (15)	8, (8)
Total electron exposure (e−/Å2)	70, (74)	77, (68)
Number of frames	140, (116)	140, (120)
Defocus range (μm)	-1 to -2	-1 to -2
Pixel size (Å)	0.835	0.835
Number of micrographs	4,882	4,559
Initial particle images (no.)	1,593,955	1,215,874
Data processing: C2 symmetry		
Final particle images (no.)	92,424	45,834
Symmetry	C2	C2
Map resolution (Å)	3.5	3.3
**Refinement**		
Initial model used (PDB code)	1WD9, 6OTC, 1N8Z	1WD9, 6OTC, 1N8Z
Symmetry	C2	C2
PDB code		
EMDB code		
Model resolution (Å)	3.5	3.3
FSC threshold	0.143	0.143
Map sharpening B factor (Å2)	-67	-100
Model composition Non-hydrogen atoms	30762	24640
Protein residues	2000	1594
Ligands	10	6
B factors (Å2)		
Protein (min/max/mean)	0.00/578.92/95.83	37.44/357.03/79.88
Ligand	0.00/52.03/33.01	53.31/103.13/74.46
RMS deviations		
Bond lengths (Å)	0.011 (12)	0.010 (8)
Bond angles (°)	1.450 (8)	1.211 (4)
Validation		
MolProbity score	0.64	0.81
Clash score	0.39	0.85
Rotamer outliers (%)	0	0.14
Ramachandran plot		
Favored (%)	98.41	97.8

Allowed (%)	1.59	2.08
Disallowed (%)	0	0.13

Supplementary Table 2. Cryo-EM data collection, processing, and refinement statistics.

Supplementary Note 1

Antibody Sequences:

Fab-hl281

Light Chain (AA):

DIQMTQSPSSLSASVGDRVTITCRASQSVSSAVAWYQQKPGKAPKLLIYSASSLYSGVPSRFSGSRSGTDFTLTISSLQPEDFATYYC
QQSYYRTLFTFGQGTKVEIKRTVAAPSVFIFPPSDSQLKSGTASVVCLLNNFYPREAKVQWKVDNALQSGNSQESVTEQDSKDSTYS
LSSTLTLSKADYEKHKVYACEVTHQGLSSPVTKSFNRGEC

Heavy Chain (AA):

EVQLVESGGGLVQPGGSLRLSCAASGFNVSYSSIHWVRQAPGKGLEWVASIYPYYGSTSYADSVKGRFTISADTSKNTAYLQMNSL
RAEDTAVYYCARQMYYWMFSKLALDYWGQGTLVTVSSASTKGPSVFPLAPSSKSTSGGTAALGCLVKDYFPEPVTVSWNSGALTS
GVHTFPAVLQSSGLYSLSSVVTVPSSSLGTQTYICNVNHKPSNTKVDKKVEPKSCDKTHT

Fab-hl364

Light Chain (AA):

DIQMTQSPSSLSASVGDRVTITCRASQSVSSAVAWYQQKPGKAPKLLIYSASSLYSGVPSRFSGSRSGTDFTLTISSLQPEDFATYYC
QQSSSSLITFGQGTKVEIKRTVAAPSVFIFPPSDSQLKSGTASVVCLLNNFYPREAKVQWKVDNALQSGNSQESVTEQDSKDSTYSL
SSTLTLSKADYEKHKVYACEVTHQGLSSPVTKSFNRGEC

Heavy Chain (AA):

EVQLVESGGGLVQPGGSLRLSCAASGFNVYSSSIHWVRQAPGKGLEWVASISSSSGSTSYADSVKGRFTISADTSKNTAYLQMNSL
RAEDTAVYYCARYSDHYYYWSYWSSWYSGLDYWGQGTLVTVSSASTKGPSVFPLAPSSKSTSGGTAALGCLVKDYFPEPVTVSWN
SGALTSGVHTFPAVLQSSGLYSLSSVVTVPSSSLGTQTYICNVNHKPSNTKVDKKVEPKSCDKTHT

Fab-hl365

Light Chain (AA):

DIQMTQSPSSLSASVGDRVTITCRASQSVSSAVAWYQQKPGKAPKLLIYSASSLYSGVPSRFSGSRSGTDFTLTISSLQPEDFATYYC
QQSSSSLVTFGQGTKVEIKRTVAAPSVFIFPPSDSQLKSGTASVVCLLNNFYPREAKVQWKVDNALQSGNSQESVTEQDSKDSTYSL
SSTLTLSKADYEKHKVYACEVTHQGLSSPVTKSFNRGEC

Heavy Chain (AA):

EVQLVESGGGLVQPGGSLRLSCAASGFNFYYSIHWVRQAPGKGLEWVASISPYSGYTSYADSVKGRFTISADTSKNTAYLQMNSLR
AEDTAVYYCARKHPGSYPFWGWALDYWGQGTLVTVSSASTKGPSVFPLAPSSKSTSGGTAALGCLVKDYFPEPVTVSWNSGALTS
GVHTFPAVLQSSGLYSLSSVVTVPSSSLGTQTYICNVNHKPSNTKVDKKVEPKSCDKTHT

Fab-hl365-E3

Light Chain (AA):

DIQMTQSPSSLSASVGDRVTITCRASQSVSSAVAWYQQKPGKAPKLLIYSASSLYSGVPSRFSGSRSGTDFTLTISSLQPEDFATYYC
QQSSSSLVTFGQGTKVEIKRTVAAPSVFIFPPSDSQLKSGTASVVCLLNNFYPREAKVQWKVDNALQSGNSQESVTEQDSKDSTYSL
SSTLTLSKADYEKHKVYACEVTHQGLSSPVTKSFNRGEC

Heavy Chain (AA):

EVQLVESGGGLVQPGGSLRLSCAASGFNF**F**YSIHWVRQAPGKGLEWVASISPY**TD**YTSYADSVKGRFTISADTSKNTAYLQMNSLRA
EDTAVYYCARKHPGSYPFWG**F**ALDYWGQGTLVTVSSASTKGPSVFPLAPSSKSTSGGTAALGCLVKDYFPEPVTVSWNSGALTSG
VHTFPAVLQSSGLYSLSSVVTVPSSSLGTQTYICNVNHKPSNTKVDKKVEPKSCDKTHT

Fab-hl365-E6

Light Chain (AA):

DIQMTQSPSSLSASVGDRVTITCRASQSVSSAVAWYQQKPGKAPKLLIYSASSLYSGVPSRFSGSRSGTDFTLTISSLQPEDFATYYC
QQS**M**SS**Q**LVTFGQGTKVEIKRTVAAPSVFIFPPSDSQLKSGTASVVCLLNNFYPREAKVQWKVDNALQSGNSQESVTEQDSKDSTY
SLSSTLTLSKADYEKHKVYACEVTHQGLSSPVTKSFNRGEC

Heavy Chain (AA):

EVQLVESGGGLVQPGGSLRLSCAASGFNFYYSIHWVRQAPGKGLEWVASISPY**TDR**TSYADSVKGRFTISADTSKNTAYLQMNSLR
AEDTAVYYCARKHPG**R**YP**N**WG**F**ALDYWGQGTLVTVSSASTKGPSVFPLAPSSKSTSGGTAALGCLVKDYFPEPVTVSWNSGALTS
GVHTFPAVLQSSGLYSLSSVVTVPSSSLGTQTYICNVNHKPSNTKVDKKVEPKSCDKTHT

Fab-mA342

Light Chain (AA):

DIQMTQSPSSLSASVGDRVTITCRASQSVSSAVAWYQQKPGKAPKLLIYSASSLYSGVPSRFSGSRSGTDFTLTISSLQPEDFATYYC
QQWSYSRLITFGQGTKVEIKRTVAAPSVFIFPPSDSQLKSGTASVVCLLNNFYPREAKVQWKVDNALQSGNSQESVTEQDSKDSTYS
LSSTLTLSKADYEKHKVYACEVTHQGLSSPVTKSFNRGEC

Heavy Chain (AA):

EVQLVESGGGLVQPGGSLRLSCAASGFNVYYSSSIHWVRQAPGKGLEWVASISSSSGYTYYADSVKGRFTISADTSKNTAYLQMNSL
RAEDTAVYYCARYSYSIYYYSGYPYYHMGLDYWGQGTLVTVSSASTKGPSVFPLAPSSKSTSGGTAALGCLVKDYFPEPVTVSWNS
GALTSGVHTFPAVLQSSGLYSLSSVVTVPSSSLGTQTYICNVNHKPSNTKVDKKVEPKSCDKTHT

Fab-mA342-c4 mutant

Light Chain (AA):

DIQMTQSPSSLSASVGDRVTITCRASQSVSSAVAWYQQKPGKAPKLLIYSASSLYSGVPSRFSGSRSGTDFTLTISSLQPEDFATYYC
QQWSYSRLATFGQGTKVEIKRTVAAPSVFIFPPSDSQLKSGTASVVCLLNNFYPREAKVQWKVDNALQSGNSQESVTEQDSKDSTY
SLSSTLTLSKADYEKHKVYACEVTHQGLSSPVTKSFNRGEC

Heavy Chain (AA):

EVQLVESGGGLVQPGGSLRLSCAASGFNVYSSSIHWVRQAPGKGLEWVASISSSSGYTYYADSVKGRFTISADTSKNTAYLQMNSL
RAEDTAVYYCARYSASIYYYSGYPAYHAGLDYWGQGTLVTVSSASTKGPSVFPLAPSSKSTSGGTAALGCLVKDYFPEPVTVSWNS
GALTSGVHTFPAVLQSSGLYSLSSVVTVPSSSLGTQTYICNVNHKPSNTKVDKKVEPKSCDKTHT

Fab-hA288

Light Chain (AA):

DIQMTQSPSSLSASVGDRVTITCRASQSVSSAVAWYQQKPGKAPKLLIYSASSLYSGVPSRFSGSRSGTDFTLTISSLQPEDFATYYC
QQYRQYNLITFGQGTKVEIKRTVAAPSVFIFPPSDSQLKSGTASVVCLLNNFYPREAKVQWKVDNALQSGNSQESVTEQDSKDSTYS
LSSTLTLSKADYEKHKVYACEVTHQGLSSPVTKSFNRGEC

Heavy Chain (AA):

EVQLVESGGGLVQPGGSLRLSCAASGFNVYYSSIHWVRQAPGKGLEWVASIYSYSGSTSYADSVKGRFTISADTSKNTAYLQMNSL
RAEDTAVYYCARYWPKMAGYWYIYRFALDYWGQGTLVTVSSASTKGPSVFPLAPSSKSTSGGTAALGCLVKDYFPEPVTVSWNSG
ALTSGVHTFPAVLQSSGLYSLSSVVTVPSSSLGTQTYICNVNHKPSNTKVDKKVEPKSCDKTHT

Fab-hA362

Light Chain (AA):

DIQMTQSPSSLSASVGDRVTITCRASQSVSSAVAWYQQKPGKAPKLLIYSASSLYSGVPSRFSGSRSGTDFTLTISSLQPEDFATYYC
QQSSYLPLFTFGQGTKVEIKRTVAAPSVFIFPPSDSQLKSGTASVVCLLNNFYPREAKVQWKVDNALQSGNSQESVTEQDSKDSTYS
LSSTLTLSKADYEKHKVYACEVTHQGLSSPVTKSFNRGEC

Heavy Chain (AA):

EVQLVESGGGLVQPGGSLRLSCAASGFNVSYYSIHWVRQAPGKGLEWVASISPYYGSTYYADSVKGRFTISADTSKNTAYLQMNSL
RAEDTAVYYCARHPYRKGYSGLDYWGQGTLVTVSSASTKGPSVFPLAPSSKSTSGGTAALGCLVKDYFPEPVTVSWNSGALTSGV
HTFPAVLQSSGLYSLSSVVTVPSSSLGTQTYICNVNHKPSNTKVDKKVEPKSCDKTHT

Supplementary note 2:

Computational methods: command lines and input files:

Example *Parameters.xml file*

```
<ROSETTASCRIPTS>
	<SCOREFXNS>
		<ScoreFunction name="beta" weights="beta_nov16" />
	</SCOREFXNS>
	<MOVERS>
		<InterfaceAnalyzerMover fixedchains="F_E_D" name="int_ddG" scorefxn="beta" />
		<MutateResidue name="mutate_residue_1" new_res="HIS" target="58F" />
		<MinMover bb="1" chi="1" name="minimize" scorefxn="beta" tolerance="0.005" />
		<RepackMinimize design_partner1="0" design_partner2="0"
interface_cutoff_distance="6.0" minimize_bb="1" minimize_sc="1" name="repack_interface"
optimize_fold_tree="1" repack_non_ala="1" repack_partner1="1" repack_partner2="1"
scorefxn_minimize="beta" scorefxn_repack="beta" />
	</MOVERS>
	<PROTOCOLS>
		<Add mover_name="mutate_residue_1" />
		<Add mover_name="repack_interface" />
		<Add mover_name="minimize" />
		<Add mover_name="int_ddG" />
	</PROTOCOLS>
</ROSETTASCRIPTS>
```

Example *shell script*

```
for name in 58
do
	filename="${name}F.xml"
	echo $filename
	for amino in ARG LYS ASP GLU SER THR ASN GLN GLY PRO ALA ILE LEU MET PHE TRP TYR VAL HIS
	do
		/usr/bin/python3 change_file.py $filename $amino
		suffix="_${name}$amino"
		/home/shared/Rosetta/main/source/bin/rosetta_scripts.linuxgccrelease -
parser:protocol $filename -in:file:s Ab365_PAD4_rm_noCA.pdb @flags.txt -database
/home/shared/Rosetta/main/database -out:path:pdb mutant_pdb/ -out:path:score mutant_score/ -
out:suffix $suffix
	done
done
```

Example *change_file.py*

```
import xml.etree.ElementTree as ET
import sys

# amino = [ALA ILE LEU MET PHE TRP TRP TYR VAL]

file_name = sys.argv[1]
amino_name = sys.argv[2]
# amino_name = amino[idx]

file = ET.parse(file_name)
root = file.getroot()

res = root.findall('MOVERS/MutateResidue')

res[0].set('new_res', amino_name)

file.write(file_name)
```

Example *flags.txt*

```
-packing
 -ex1
 -ex1aro
 -extrachi_cutoff 0
 -ex2
-nstruct 5
-overwrite
-mute core.util.prof
```

```
-mute core.io.database
-corrections::beta_nov16
```

Example script for *Rosetta Antibody design of CDR L3*

```
antibody_designer.linuxgccrelease -s PAD4-FAB_relaxed_renumbered_min_no_calcium.pdb -
graft_design_cdrs L1 L3 -seq_design_cdrs L1 L3 -light_chain kappa -mc_optimize_dG -do_dock -
use_epitope_constraints -nstruct 1000 -scorefile_format json -out:prefix PAD4_
```

OR

```
mpirun -np 16 antibody_designer.mpi.linuxgccrelease @ common
```

Example *common file for mpirun*

```
#Input
-s relaxed_calcium.pdb
#-ignore_unrecognized_res
#-ignore_zero_occupancy false
#-load_PDB_components false

#design
-graft_design_cdrs  L3
-seq_design_cdrs L3
-light_chain kappa

#number of designs
-nstruct 1000

-random_start
-allow_omega_mismatches_for_north_clusters

#cdr instructions
#-cdr_instructions cdr_instructions.txt

#optimize
-mc_optimize_dG
#-mc_total_weight .001
#-mc_interface_weight .999
#-mintype relax

#Output
-scorefile_format json
#-pdb_comments
#-skip_connect_info
-out:prefix PAD4

#docking & constraints
#-do_dock
#-use_epitope_constraints

#Rotamers/packing (Generally recommended, but will slow us down here)
-ex1
-ex2
-use_input_sc

#RAbD Options for speed
-outer_cycle_rounds 25
-inner_cycle_rounds 2
```
