# Supplementary material for: Antibody discovery identifies regulatory mechanisms of protein arginine deiminase 4
Source: Nat Chem Biol. 2024 Feb 2;20(6):742–50. doi: 10.1038/s41589-023-01535-8 (PMC11142921; doi:10.1038/s41589-023-01535-8)
Supplement: Supplementary file 2 — Reporting Summary [file 41589_2023_1535_MOESM2_ESM.pdf]

Corresponding author(s): James A Wells

Last updated by author(s): Dec 6, 2023

Reporting Summary

Nature Portfolio wishes to improve the reproducibility of the work that we publish. This form provides structure for consistency and transparency in reporting. For further information on Nature Portfolio policies, see our Editorial Policies and the Editorial Policy Checklist.

Statistics

For all statistical analyses, confirm that the following items are present in the figure legend, table legend, main text, or Methods section.

n/a	Confirmed	
☐	☒	The exact sample size (*n*) for each experimental group/condition, given as a discrete number and unit of measurement
☐	☒	A statement on whether measurements were taken from distinct samples or whether the same sample was measured repeatedly
☒	☐	The statistical test(s) used AND whether they are one- or two-sided<br>*Only common tests should be described solely by name; describe more complex techniques in the Methods section.*
☒	☐	A description of all covariates tested
☒	☐	A description of any assumptions or corrections, such as tests of normality and adjustment for multiple comparisons
☐	☒	A full description of the statistical parameters including central tendency (e.g. means) or other basic estimates (e.g. regression coefficient) AND variation (e.g. standard deviation) or associated estimates of uncertainty (e.g. confidence intervals)
☒	☐	For null hypothesis testing, the test statistic (e.g. *F*, *t*, *r*) with confidence intervals, effect sizes, degrees of freedom and *P* value noted<br>*Give P values as exact values whenever suitable.*
☒	☐	For Bayesian analysis, information on the choice of priors and Markov chain Monte Carlo settings
☒	☐	For hierarchical and complex designs, identification of the appropriate level for tests and full reporting of outcomes
☒	☐	Estimates of effect sizes (e.g. Cohen's *d*, Pearson's *r*), indicating how they were calculated

Our web collection on statistics for biologists contains articles on many of the points above.

Software and code

Policy information about availability of computer code

Data collection	ForteBio Octet Data Aquisition (v 12.0.2.11) was used for biolayer interferometry (BLI) aquisition. Image Studio Software (v 5.2) and Image Lab (v 5.0) was used for western blot scanning. Tecan i-control (v 3.4.3.0) was used to collect plate reader scans.
Data analysis	ForteBio Data Analysis (v 12.0) was used for BLI data analysis. Fiji was used for western blot quantification. GraphPad Prism (v 9.2.0) was used for statistics and data analysis. cyoSPARC software was used for cryoEMG analysis. Refinement and class sorting were performed in cisTEM (v 1.0.0). Models were refined with Phenix Real-Space Refinement.

For manuscripts utilizing custom algorithms or software that are central to the research but not yet described in published literature, software must be made available to editors and reviewers. We strongly encourage code deposition in a community repository (e.g. GitHub). See the Nature Portfolio guidelines for submitting code & software for further information.

Data

Policy information about availability of data

All manuscripts must include a data availability statement. This statement should provide the following information, where applicable:

- Accession codes, unique identifiers, or web links for publicly available datasets
- A description of any restrictions on data availability
- For clinical datasets or third party data, please ensure that the statement adheres to our policy

Cryo-EM structural data are deposited in the Protein Data Bank and Electron Microscopy Data Bank. The hPADI4/hA362 complex is deposited as PDB 8SMK and

EMD-40589. The hPADI4/hI365 complex is deposited as PDB 8SML and EMD-40590. The crystal structure of hPADI4 from PDB 1WDA was used for dimer interface modeling and for reference against our inhibitor-bound PADI4 cryo-EM complexes. Source data are provided with this paper. Any additional information is available upon request.

Human research participants

Policy information about studies involving human research participants and Sex and Gender in Research.

Reporting on sex and gender	Study did not involve human research.
Population characteristics	Study did not involve human research.
Recruitment	Study did not involve human research.
Ethics oversight	Study did not involve human research.

Note that full information on the approval of the study protocol must also be provided in the manuscript.

Field-specific reporting

Please select the one below that is the best fit for your research. If you are not sure, read the appropriate sections before making your selection.

☒ Life sciences ☐ Behavioural & social sciences ☐ Ecological, evolutionary & environmental sciences

For a reference copy of the document with all sections, see nature.com/documents/nr-reporting-summary-flat.pdf

Life sciences study design

All studies must disclose on these points even when the disclosure is negative.

Sample size	Statistical methods to predetermine sample size were not conducted. Sample size of data collected were chosen such that statistical significant were clear.
Data exclusions	No data were excluded.
Replication	All samples were tested in technical triplicate and all attempts at biological replication were successful.
Randomization	Randomization is not relevant as we did not use experimental groups.
Blinding	Blinding is not relevant as we did not use experimental groups.

Reporting for specific materials, systems and methods

We require information from authors about some types of materials, experimental systems and methods used in many studies. Here, indicate whether each material, system or method listed is relevant to your study. If you are not sure if a list item applies to your research, read the appropriate section before selecting a response.

Materials & experimental systems

n/a	Involved in the study	
☐	☒	Antibodies
☐	☒	Eukaryotic cell lines
☒	☐	Palaeontology and archaeology
☒	☐	Animals and other organisms
☒	☐	Clinical data
☒	☐	Dual use research of concern

Methods

n/a	Involved in the study	
☒	☐	ChIP-seq
☒	☐	Flow cytometry
☒	☐	MRI-based neuroimaging

Antibodies

Antibodies used	hA288, hI280, hA362, hI364, hI365, hI365-E3, hI365-E6, mA342, mA342-c4
Validation	hA288, hI280, hA362, hI364, hI365, hI365-E3, hI365-E6, mA342, and mA342-c4 were generated in-house using a human or mouse

Validation	Fab-phage synthetic library and validated via Sanger sequencing of DNA plasmids. Affinity was validated by BLI.<br><br>Rabbit anti-citrullinated H3 (abcam 5103), 1:1000 WB dilution. Validation accessible on manufacturer's website.<br>Goat anti-rabbit HRP (Cell Signaling Technology 7074), 1:10000 WB dilution. Validation accessible on manufacturer's website.

Eukaryotic cell lines

Policy information about cell lines and Sex and Gender in Research

Cell line source(s)	Flp-in HEK293 cells were obtained from ThermoFisher (Cat. R75007) and engineered to express PAD4.
Authentication	Flp-in HEK293 cells were validated by supplier, Flp-in HEK293 cells expressing fluorescent PAD4 were validated by fluorescence microscopy and subsequently sorted using fluorescence-activated cell sorting (FACS).
Mycoplasma contamination	Cell lines tested negative for mycoplasma contamination.
Commonly misidentified lines<br>(See ICLAC register)	No commonly misidentified lines were used.
