# Supplementary figures and images for: Antibody discovery identifies regulatory mechanisms of protein arginine deiminase 4
Source: Nat Chem Biol. 2024 Feb 2;20(6):742–50. doi: 10.1038/s41589-023-01535-8 (PMC11142921; doi:10.1038/s41589-023-01535-8)

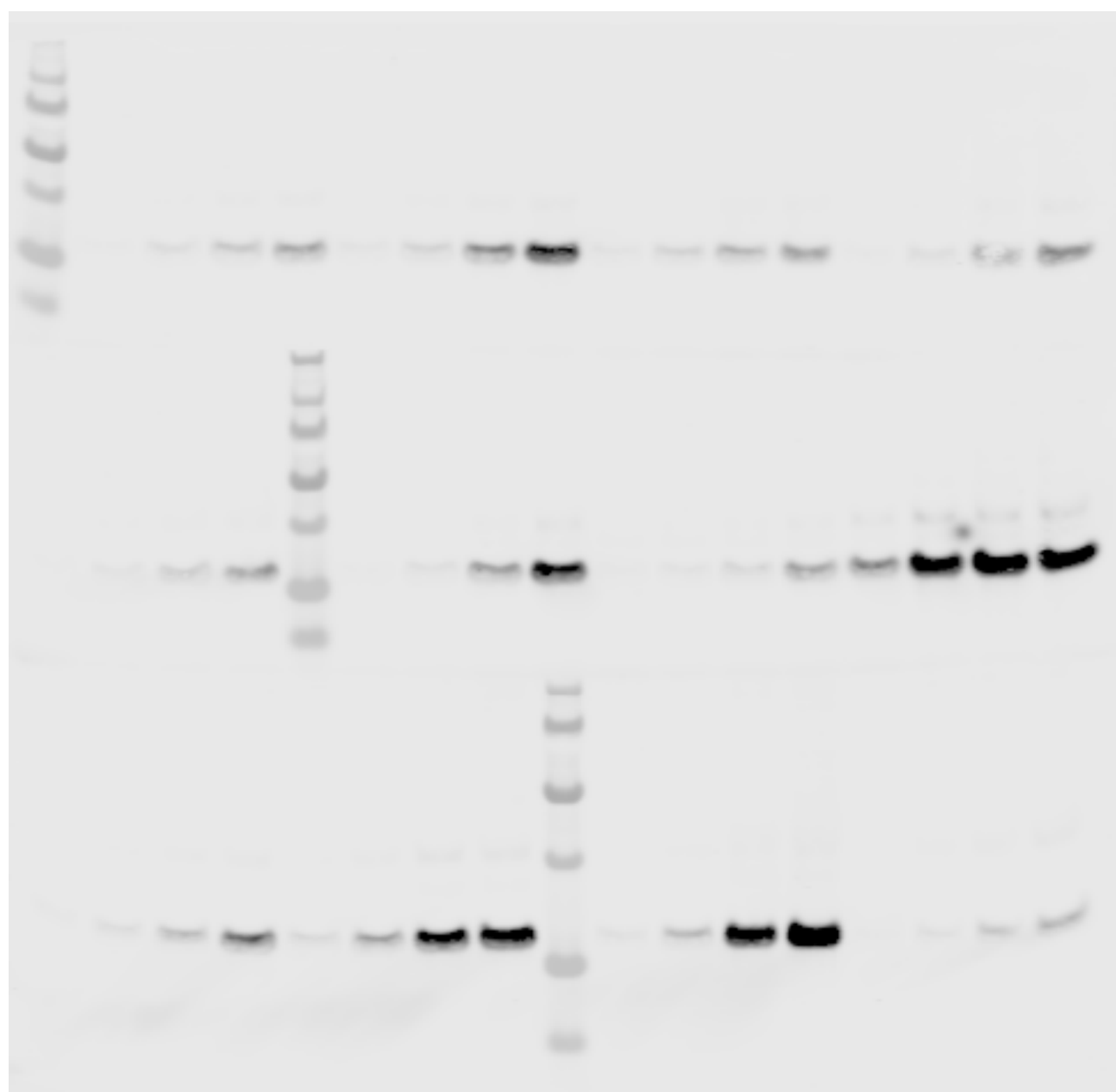

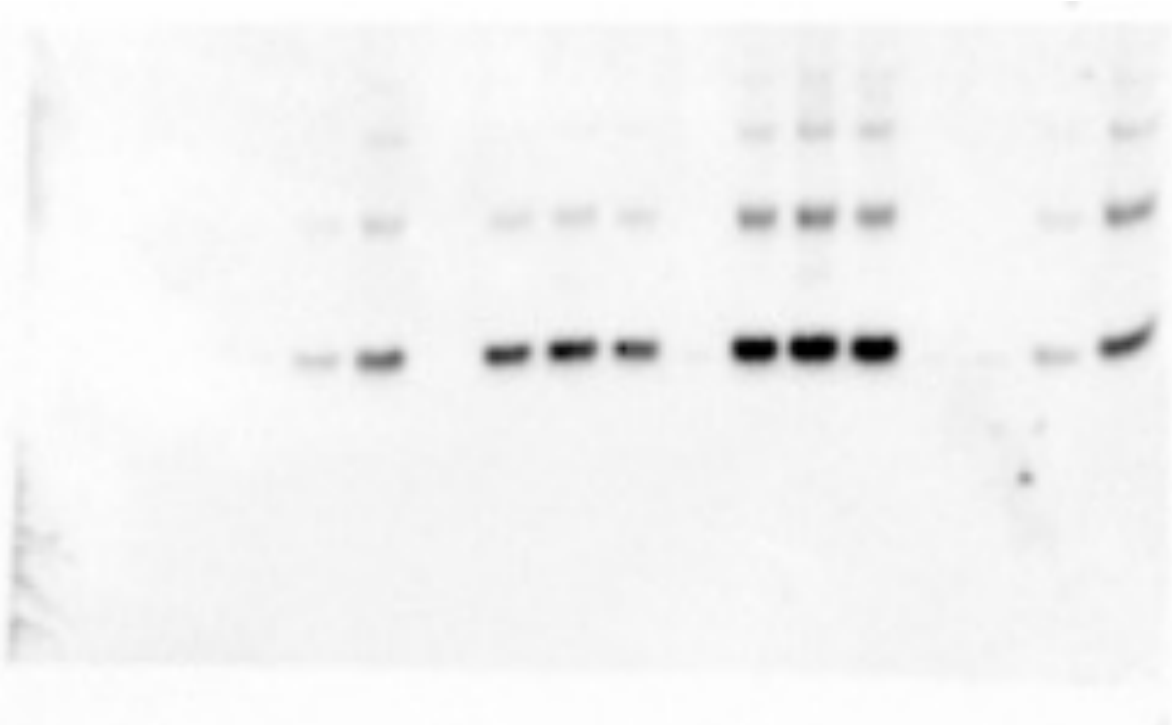

Supplement: Supplementary file 3 — Source data, unprocessed western blots. [file 41589_2023_1535_MOESM3_ESM.zip › Fig1_ cit_H3_WB_humanmouse-combined.pdf]

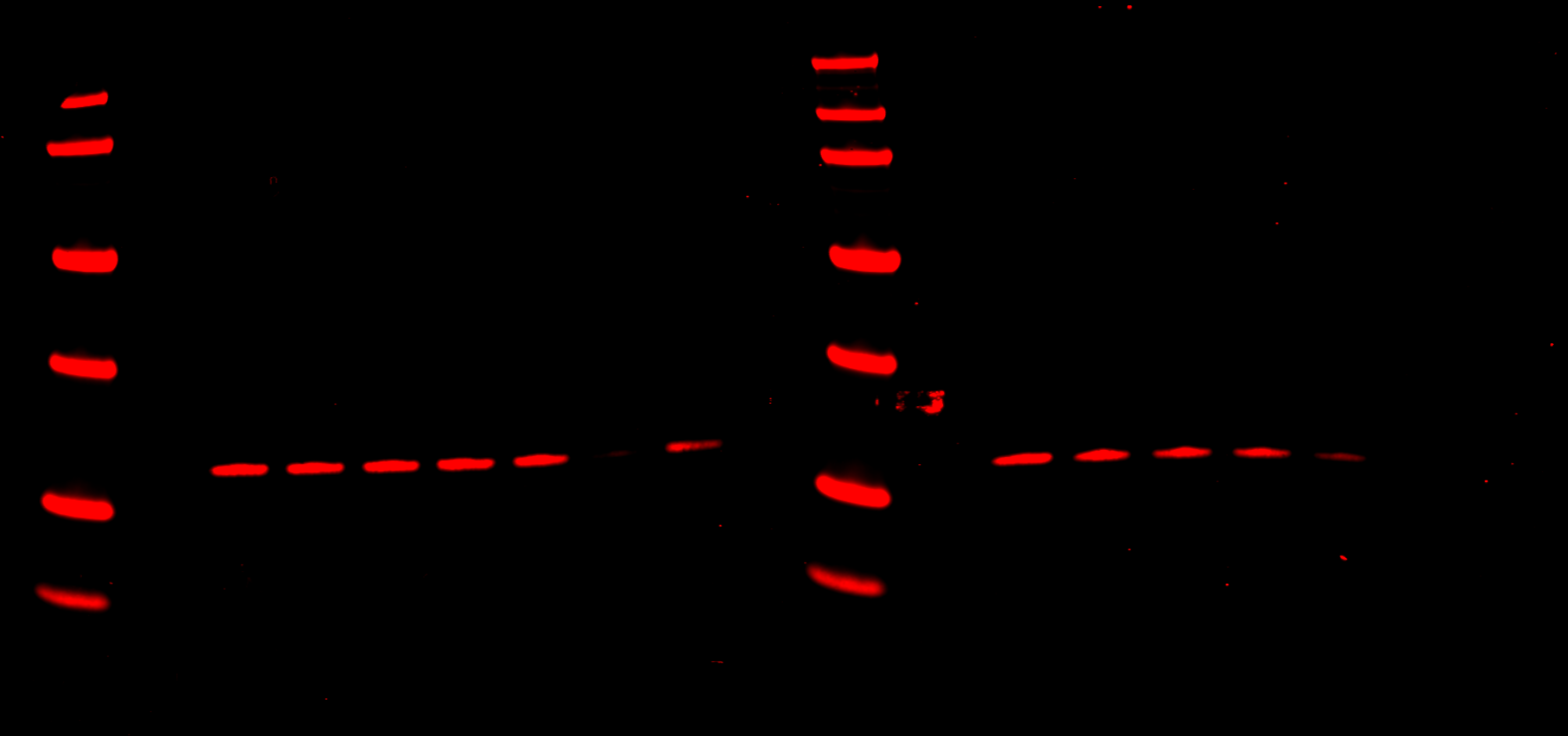

Supplement: Supplementary file 5 — Source data, unprocessed western blots. [file 41589_2023_1535_MOESM5_ESM.zip › Fig5_IC50_WB_rep3.tif]

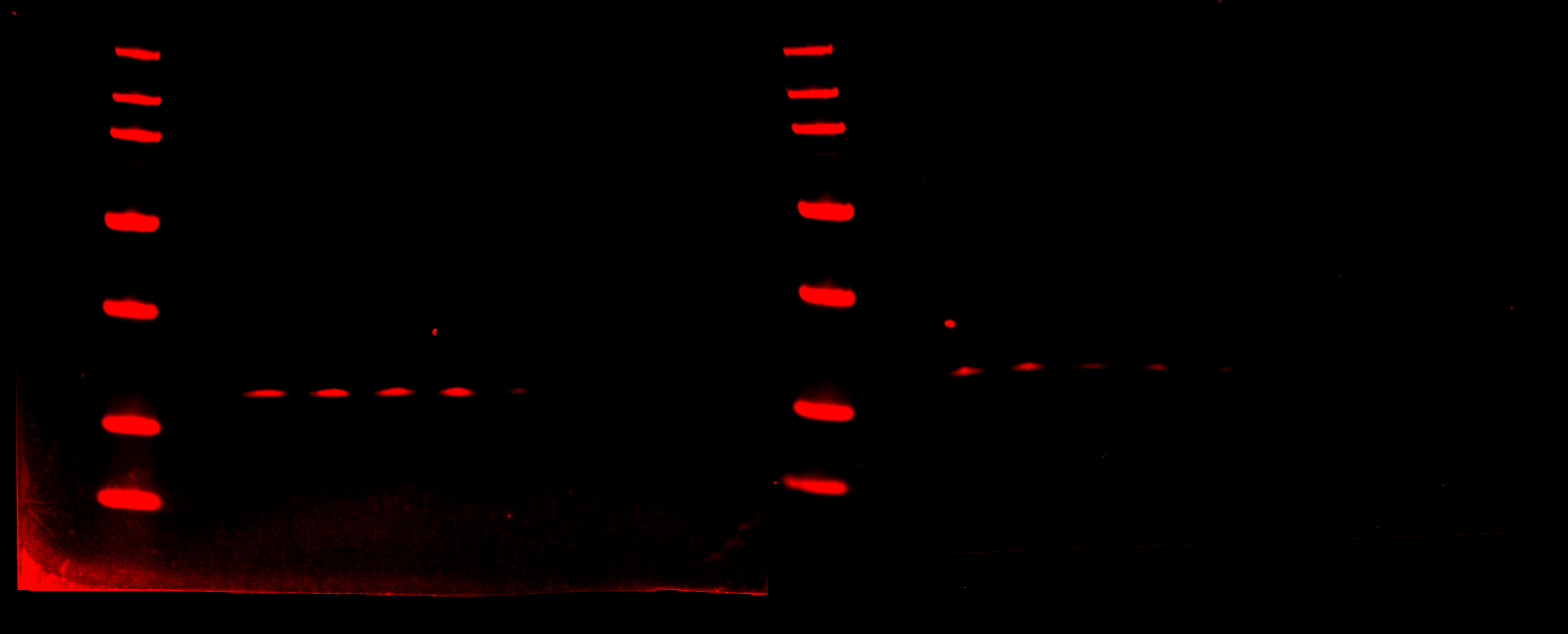

Supplement: Supplementary file 5 — Source data, unprocessed western blots. [file 41589_2023_1535_MOESM5_ESM.zip › Fig5_IC50_WB_rep2.tif]

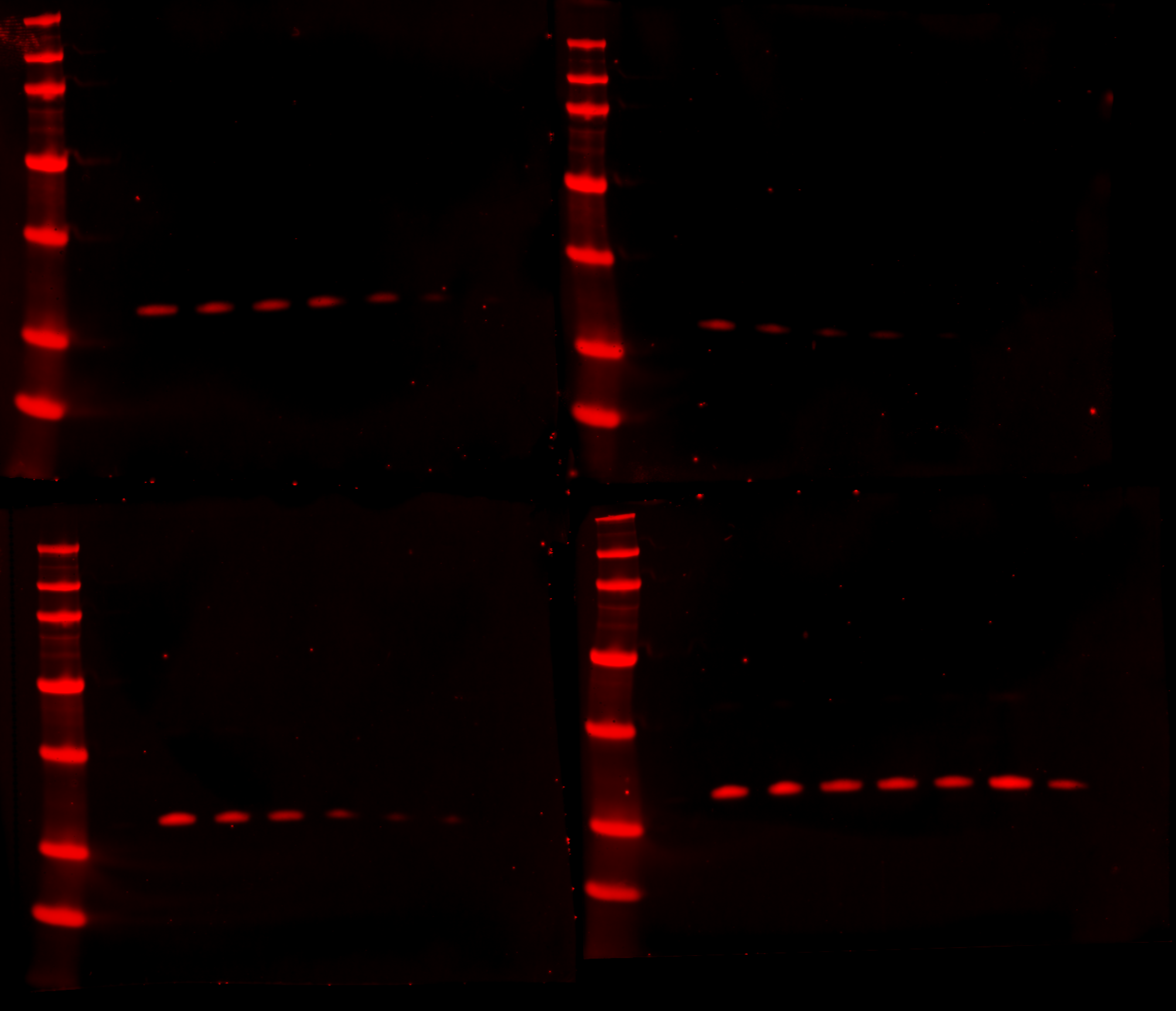

Supplement: Supplementary file 5 — Source data, unprocessed western blots. [file 41589_2023_1535_MOESM5_ESM.zip › Fig5_IC50_WB_rep1.tif]

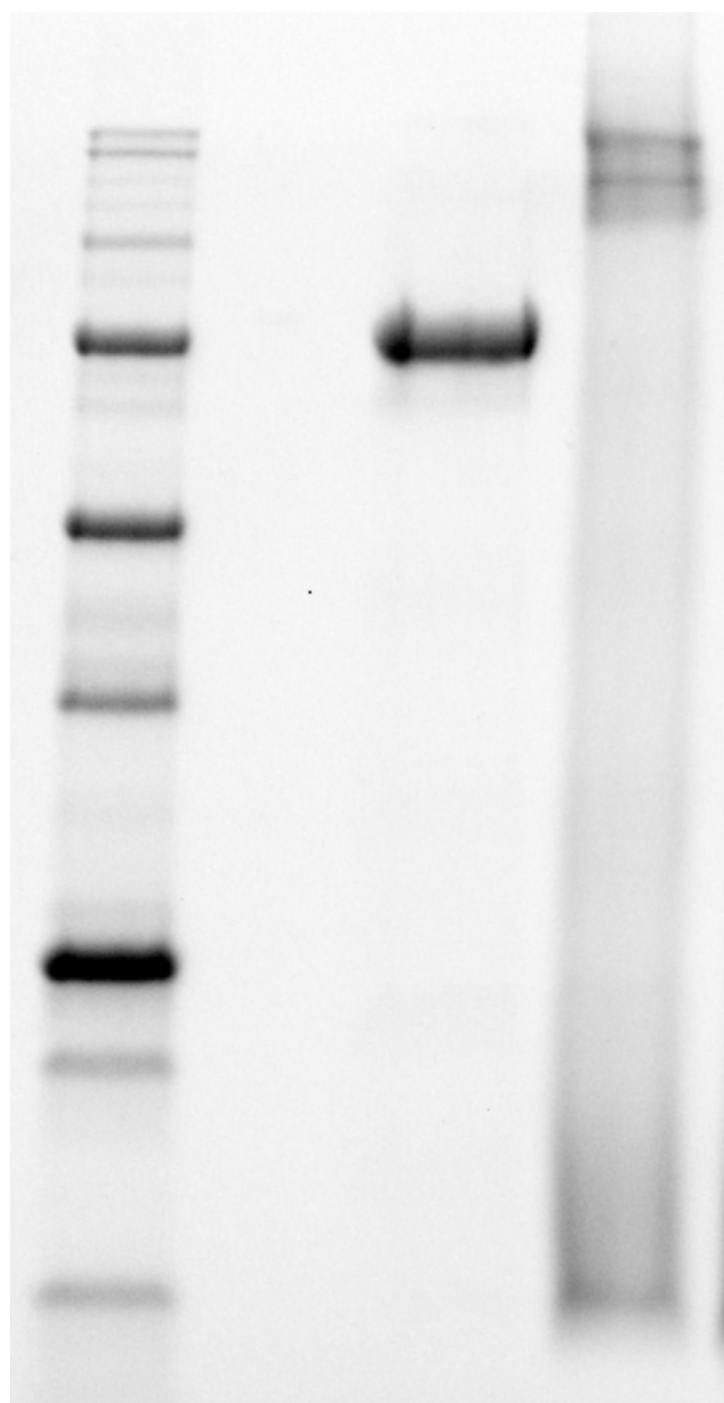

Supplement: Supplementary file 6 — Source data, unprocessed SDS–PAGE gels. [file 41589_2023_1535_MOESM6_ESM.zip › ExtendedData1B_PAD4antigen.pdf]

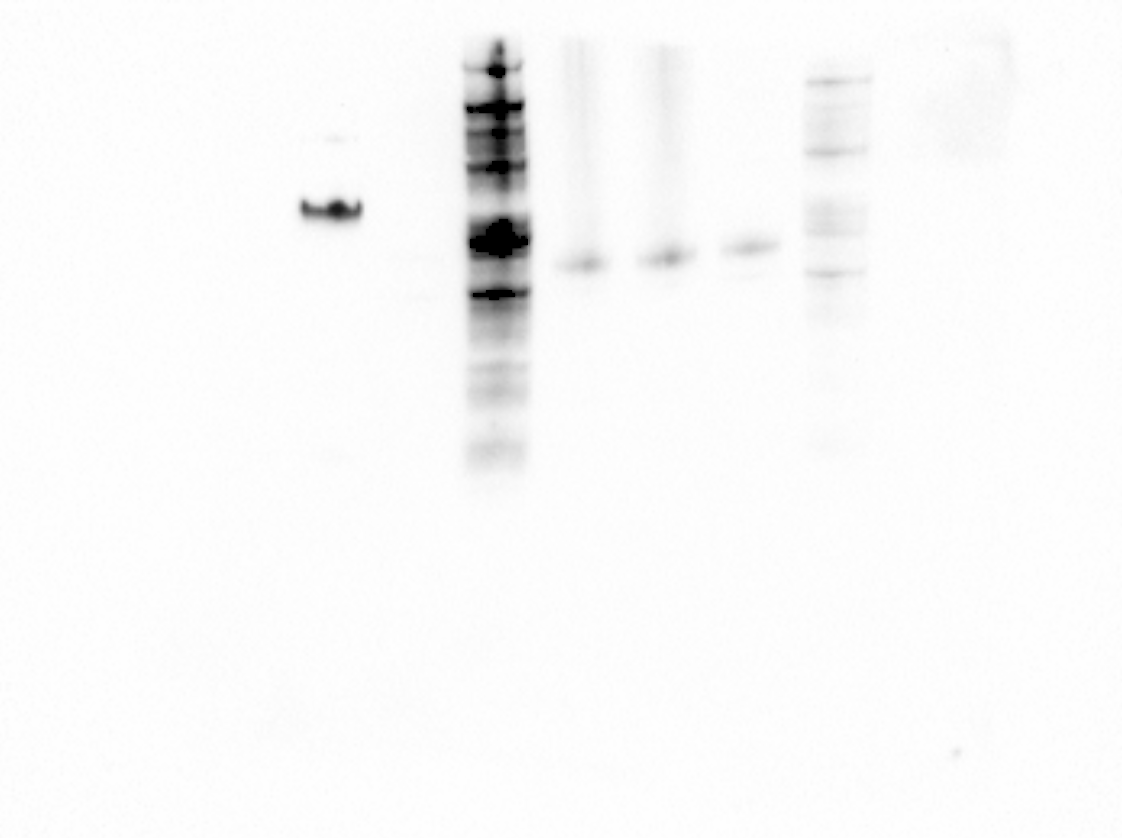

Supplement: Supplementary file 12 — Source data, unprocessed western blots. [file 41589_2023_1535_MOESM12_ESM.zip › ExtendedData8B_cit-ome_WB.tif]

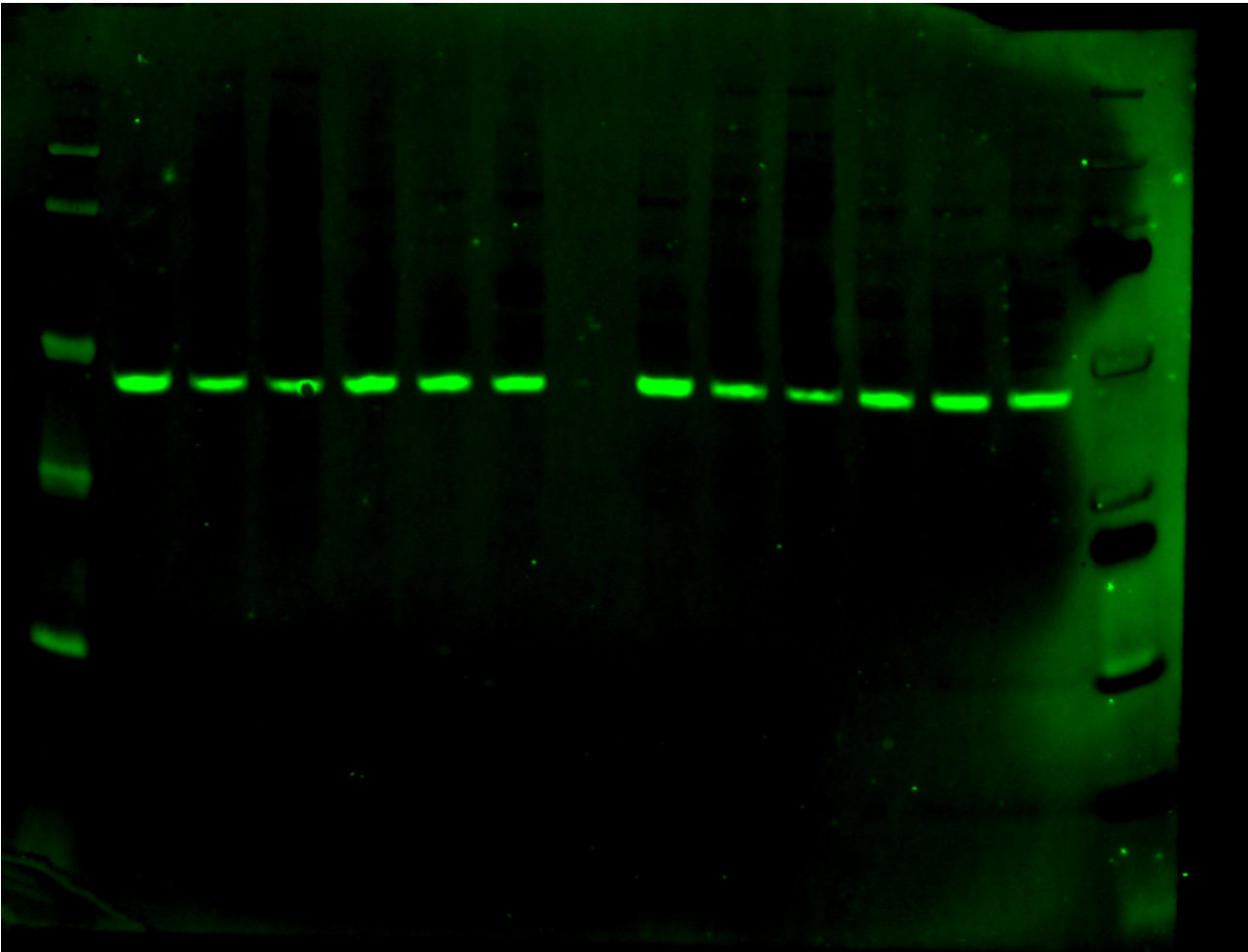

Supplement: Supplementary file 12 — Source data, unprocessed western blots. [file 41589_2023_1535_MOESM12_ESM.zip › ExtendedData8B_cit-ome_actin_WB.pdf]
